# Electro-optical mechanically flexible coaxial microprobes for minimally invasive interfacing with intrinsic neural circuits

**DOI:** 10.1038/s41467-022-30275-x

**Published:** 2022-06-07

**Authors:** Spencer Ward, Conor Riley, Erin M. Carey, Jenny Nguyen, Sadik Esener, Axel Nimmerjahn, Donald J. Sirbuly

**Affiliations:** 1 Department of Electrical and Computer Engineering, University of California, San Diego, La Jolla, CA 92093 USA; 2 Department of Nanoengineering, University of California, San Diego, La Jolla, CA 92093 USA; 3 Waitt Advanced Biophotonics Center, Salk Institute for Biological Studies, La Jolla, CA 92037 USA; 4 Materials Science and Engineering, University of California, San Diego, La Jolla, CA 92093 USA

**Keywords:** Neural circuits, Biomedical engineering

## Abstract

Central to advancing our understanding of neural circuits is developing minimally invasive, multi-modal interfaces capable of simultaneously recording and modulating neural activity. Recent devices have focused on matching the mechanical compliance of tissue to reduce inflammatory responses. However, reductions in the size of multi-modal interfaces are needed to further improve biocompatibility and long-term recording capabilities. Here a multi-modal coaxial microprobe design with a minimally invasive footprint (8–14 µm diameter over millimeter lengths) that enables efficient electrical and optical interrogation of neural networks is presented. In the brain, the probes allowed robust electrical measurement and optogenetic stimulation. Scalable fabrication strategies can be used with various electrical and optical materials, making the probes highly customizable to experimental requirements, including length, diameter, and mechanical properties. Given their negligible inflammatory response, these probes promise to enable a new generation of readily tunable multi-modal devices for long-term, minimally invasive interfacing with neural circuits.

## Abstract (web summary)

The authors demonstrate a compact multi-modal electro-optical coaxial microprobe design that offers small cross-sectional dimensions and tunable lengths for minimally invasive interfacing with intrinsic neural circuits.

## Introduction

Microelectrode recordings are the gold standard for measuring individual neurons’ activity at high temporal resolution in any nervous system region and are central to defining the role of neural circuits in controlling behavior. Microelectrode arrays, such as the Utah or Michigan arrays, have allowed tracking of distributed neural activity with millisecond precision^1,2^. However, their large footprint and rigidity lead to tissue damage and inflammation that hamper long-term recordings^3,4^. State-of-the-art Neuropixel and carbon fiber probes have improved on these previous devices by increasing electrode density and reducing probe dimensions and rigidity^5–7^. Although these probes have advanced the field of neural interfacing, next-generation devices should enable targeted stimulation in addition to colocalized electrical recordings^3,8^. Optogenetic techniques enable high-speed modulation of cellular activity through targeted expression and activation of light-sensitive opsins^9^. However, given the strong light scattering and high absorption properties of neural tissue, optogenetic interfacing with deep neural circuits typically requires the implantation of large-diameter rigid fibers, which can make this approach more invasive than its electrical counterpart^10–12^.

The ideal neural probe would combine optical and electrical modes while maintaining small cross-sectional dimensions and tunable lengths. The ability to bi-directionally interface with genetically defined neuron types and circuits is key to ultimately being able to understand how the nervous system computes and controls behavior. It is also fundamental for determining the mechanistic basis of sensorimotor disorders, defining how circuit activity is affected by injury, and how it might be restored or facilitated. Approaches to integrating optical and electrical modalities have ranged from adding fiber optics to existing Utah arrays to the Optetrode or other integrated electro-optical coaxial structures^13–17^. These technologies have shown great promise for simultaneous electrical recordings and optical stimulation in vivo. However, the need to reduce the device footprint to minimize immune responses for long-term recordings is still present^3,18–21^.

In this work, we present, to the best of our knowledge, the smallest multi-modal coaxial neural probe with a low impedance electrical channel surrounding a small central fiber optic core. These electro-optical mechanically flexible (EO-Flex) probes can be fabricated with diameters as small as 8 µm and lengths up to several millimeters using microfiber optic waveguide cores or even smaller diameters with nanofiber optic cores. They can also be bonded directly to single-mode fibers (SMFs) to create detachable, low-loss optical interfaces that can be directly connected to standard optogenetic hardware. The EO-Flex probes’ simultaneous electrical recording and optical stimulation performance are demonstrated in the mouse brain. Our experiments show that the porous metal electrical channel provides excellent recording ability even with the probe’s small size. The low source-to-tip optical losses of <10 dB allow robust optogenetic stimulation in transgenic or virally transduced mice expressing opsins in target cells. Implant studies show minimal immune responses, suggesting that the fully customizable probe and future high-density arrays should enable long-term interfacing with minimal disturbance to the surrounding neural tissue.

## Results

**Fig. 1:**
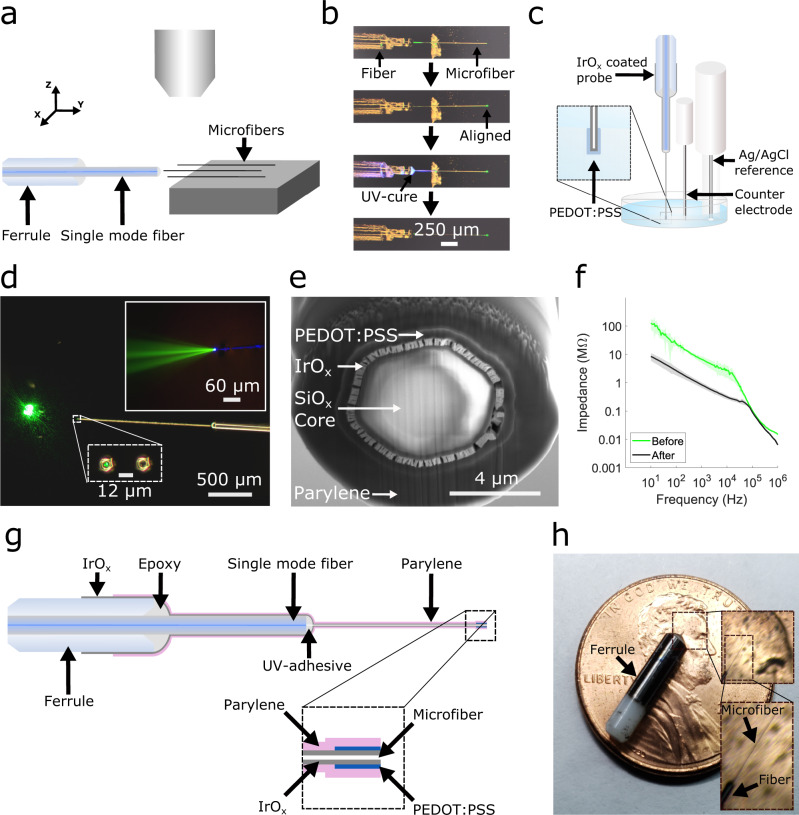
Fabrication of implantable EO-Flex probes along with optical and electrical characterization. **a** Silica microfibers of defined length are positioned on a silicon substrate to allow a single-mode fiber (SMF)-loaded ferrule to bond to the microfiber. **b** (from top to bottom) Photographs show the active alignment and bonding process of coupling the microfiber to the SMF. **c** Schematic of the electrodeposition setup for depositing poly(3,4-ethylenedioxythiophene) polystyrene sulfonate (PEDOT:PSS) after the sputtering of iridium oxide (IrO_x_). **d** Optical image of the light output of a probe from the side as the light reflects from a mirror, and from the cleaved end-facet (zoom-in insets) with and without laser light. (inset) Fluorescence image capturing the cone angle of the probe after submerging it in a dye solution and launching blue (442 nm) light into the probe. Micrographs were generated over a couple of experiments. **e** Cross-sectional electron micrograph of an EO-Flex probe after milling the end showing the exposed conductive rings along with the optical glass (SiO_x_) core. Multiple electron micrographs were recorded for similar probes resulting in similar properties. **f** EIS data for milled probes with (black line) and without (green line) the PEDOT:PSS cladding. Average impedance is shown with the lightly shaded area representing one standard deviation for *n* = 4 probes. **g** A cross-sectional view of the probe showing its various cladding layers. **h** Photograph of a completed EO-Flex probe with a zoom-in of the microfiber tip region. For scaling strategies, see Fig. S16.

EO-Flex probes were fabricated using micro- and nanofiber optical cores (see Methods). Here, we will focus on mass-producible silica microfibers as the core that enables probes with lengths surpassing 3 mm while maintaining a diameter of <12 µm (Fig. S16a). However, the fabrication protocol is general and can be used with other optical cores, including subwavelength metal oxide nanofiber waveguides, to produce ultra-miniaturized probes (Fig. S3). To enable efficient coupling to optogenetic hardware, the microfibers were first placed on a silicon substrate, with one end of the fiber protruding the edge of the substrate, and then butt-coupled to a cleaved SMF (Fig. 1a). We used active alignment to maximize mode overlap between the microfiber and SMF. The coupling was locked in using a UV-curable optical adhesive droplet on the end of the SMF (Fig. 1b).

To create a robust detachable interface for in vivo testing, the SMF was inserted into a ceramic ferrule. The distal end of the ferrule assembly was machine polished to allow the coupling to a patch cable (Fig. 1g, h). Other interface designs for different applications are conceivable (Fig. S16). To form a low noise electrically conductive layer around the probe tip, a 379 ± 43 nm layer of iridium oxide (IrO_x_) was sputtered on the microfibers followed by a 362 ± 137 nm electrochemically deposited layer of poly(3,4-ethylenedioxythiophene) polystyrene sulfonate (PEDOT:PSS) (Fig. 1c)^22^. The porous nature of IrO_x_ allowed better adhesion of the conductive PEDOT:PSS layer and enhances the overall electrical performance of the probe. The probe was passivated with 1.76 ± 0.16 µm of Parylene-C to electrically isolate the probe and provide a biocompatible surface (Fig. S2). In order to expose the electrical and optical surfaces, a focused ion beam was used to cleave off the tip (Fig. 1e; see Supporting Information). Figure 1g displays the final probe design, and Fig. 1h shows a photograph of a completed probe. Through the combination of IrO_x_ and PEDOT:PSS, electrical impedances of <1 MΩ at 1 kHz were achieved from electrode areas of <15 µm^2^ (Fig. 1f).

The probes’ optical properties were first assessed by imaging the output cone angle in a dye solution (Fig. 1d, inset), which showed a divergence angle of 10–15°. Importantly, after the cladding layers are placed on the probe, no detectable scattering light is observed from the microfiber/SMF interface (Fig. 1d) as compared to the pre-cladding probe (Fig. 1b). The optical losses between a laser-coupled patch cable and the EO-Flex output were quantified using three different wavelengths (473, 543, 600 nm) with all devices showing <7 dB (*n* = 4). These values match up well with simulated results for an ~2 µm mode misalignment in the ferrule sleeve (Fig. S1). Electrochemical impedance spectroscopy (EIS) was performed on the probes while submersed in a 1x phosphate-buffered saline (PBS). All probes fabricated and tested showed an average electrical impedance of 844 ± 179 kΩ at 1 kHz (*n* = 4; Fig. 1f and Fig. S2a–d) after cladding deposition and milling the tip, compared to >10 MΩ before PEDOT deposition.

**Fig. 2:**
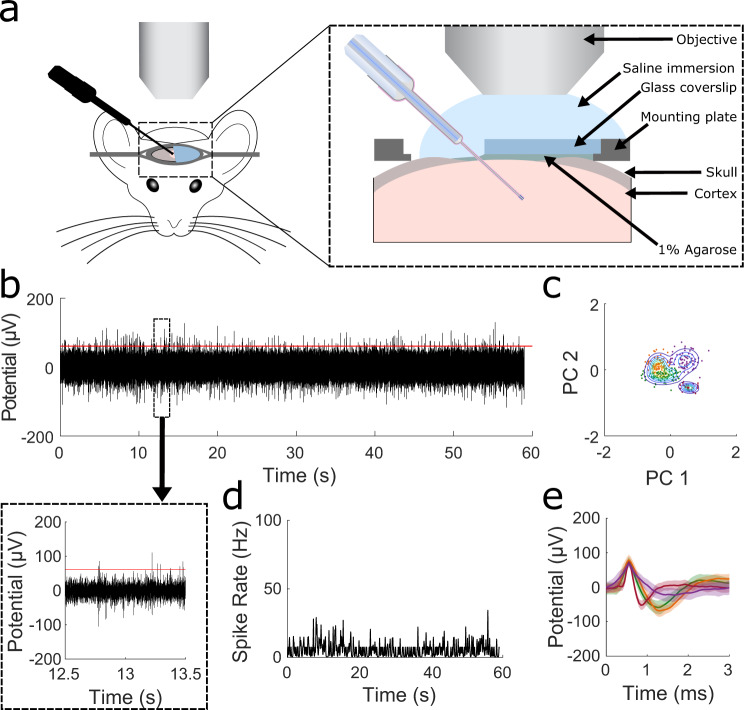
Extracellular neural recordings in the cortex of live mice using the EO-Flex probes. **a** Schematic showing the setup used for visually guided electrical measurements. Two-photon imaging of the probe in relation to fluorescently labeled cells (see Methods) was used to track its movement through tissue and optimize the recording position. (inset) Zoom-in cross-sectional view of the surgical preparation for simultaneous imaging and electrical recordings. **b** Example EO-Flex recording showing spontaneous neural activity in cortical layer 2/3 (depth = 250 µm) of an isoflurane-anesthetized mouse. The threshold (red line) defining a spike was set to ${{{{{\rm{Threshold}}}}}}=4* {{{{{\rm{median}}}}}}\left(\frac{\left|{{{{{\rm{Recording}}}}}}\right|}{0.675}\right)$ based on published literature^36^. (boxed region) A one-second excerpt from the recording shows multi-unit activity. **c** Principal component (PC) analysis plot of the waveform clustering using established clustering methods^37^. **d** Spiking rate over the 1-min recording shown in (**b**) was calculated using a Bayesian kernel estimation. **e** Average waveforms (solid lines) along with one standard deviation (shaded regions) for four clusters determined by PCA from the recording in (**b**).

To confirm that EO-Flex probes allow high-sensitivity electrical measurements in vivo, we performed simultaneous extracellular recordings and two-photon imaging in the cortex of isoflurane-anesthetized mice with fluorescently labeled cells (see Supporting Information)^23,24^. This approach enabled the monitoring of insertion and targeted movement of the probe through the tissue (Fig. 2a). Probes readily penetrated the exposed dura with minimal buckling when using water immersion (Video S1) and reached target regions in optically accessible cortical layer 2/3 (Fig. S12 and Video S2). When mounted to a three-axis micromanipulator, fine adjustment of the probe tip’s lateral position, once inside the tissue, was feasible to optimize the signal-to-noise ratio and target individual neurons. However, the lateral movement was typically limited to <30 µm. Using this approach, we acquired endogenous multi- and single-unit activity (Fig. 2b). Principle component analysis (PCA) and Gaussian clustering of electrical recordings were used to determine the number of distinct units (Fig. 2c). Spiking rates were calculated using a Bayesian Adaptive Kernel Smoother (BAKS) algorithm applied to the full duration of the recording (Fig. 2d)^25^. Figure 2b–e shows a representative recording. Using an ~1 mm-long EO-Flex probe, we also obtained electrical recordings from deeper cortical areas up to the probes’ maximum length. The electrical signature of these recordings suggests that all cortical layers can be accessed (Fig. S4).

**Fig. 3:**
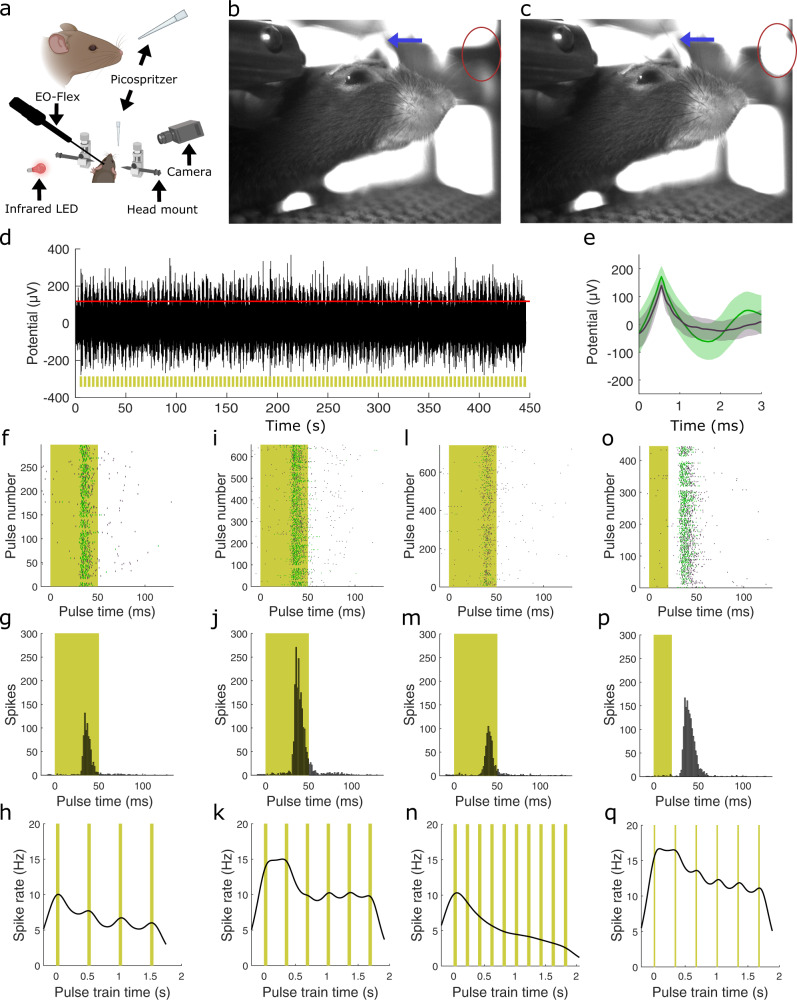
Measurement of whisker stimulation-induced sensory activity in the barrel cortex of awake head-restrained mice on a spherical treadmill using the EO-Flex probes. **a** Schematic of the experimental setup. Whiskers were deflected by air puff stimuli delivered through a micropipette connected to a function generator-controlled pressure system (Picospritzer). The function generator also controlled an infrared LED for analog and video data synchronization. **b** Video frame showing the resting animal before whisker deflection. The blue arrow indicates an example whisker. The red circle indicates the infrared LED’s location. **c** Video frame showing deflection of the indicated whisker during air puff delivery. **d** Example EO-Flex recording (black) during 3 Hz whisker stimulation (yellow). **e** Corresponding spike sorted average waveforms with one standard deviation shaded. Different neural waveforms are marked with different colors. **f**–**h** Raster plot showing whisker stimulus-evoked activity for a 2 Hz stimulation frequency (50 ms pulse width) (**f**), peristimulus time histogram (**g**), and BAKS estimation of firing frequency (**h**). **i**–**k** Raster plot (**i**), peristimulus time histogram (**j**), and BAKS estimation for a 3 Hz stimulation (50 ms pulse width). **l**–**n** Raster plot (**l**), peristimulus time histogram (**m**), and BAKS estimation (**n**) for 5 Hz stimulation (50 ms pulse width). **o**–**q** Raster plot (**o**), peristimulus time histogram (**p**), and BAKS estimation (**q**) for 3 Hz stimulation (20 ms pulse width). Panel (**a**) created with Biorender.com.

Next, to demonstrate the EO-Flex probes’ ability to record peripherally evoked activity, we inserted them into the barrel cortex, which receives sensory input from whiskers on the opposite side of the body. While advancing the probe into the brain, we periodically deflected the whiskers using air puffs while the animal was still under isoflurane anesthesia. Once the correlated activity was observed, probe position (~900 µm insertion) was locked in place and anesthesia was turned off. Measurements commenced 30–60 min after the animals began to walk. Video recordings were used to verify air puff-mediated whisker deflections and record spontaneous whisking behavior under infrared illumination (Fig. 3a–c). Additionally, mouse locomotor activity was recorded using an optical encoder attached to the spherical treadmill on which the animal was positioned. Whiskers were deflected using various pulse frequencies (2, 3, and 5 Hz) and widths (20 and 50 ms) (Fig. 3f–q), leading to stimulus-dependent increases in spike rate, which were most pronounced during periods of rest (i.e., in the absence of spontaneous whisking). To further corroborate the probe’s positioning in the barrel cortex, we conducted EO-Flex probe-mediated electrical stimulations (0–300 µA biphasic pulses at 100 Hz), resulting in current amplitude-dependent whisker deflections (Fig. S5).

**Fig. 4:**
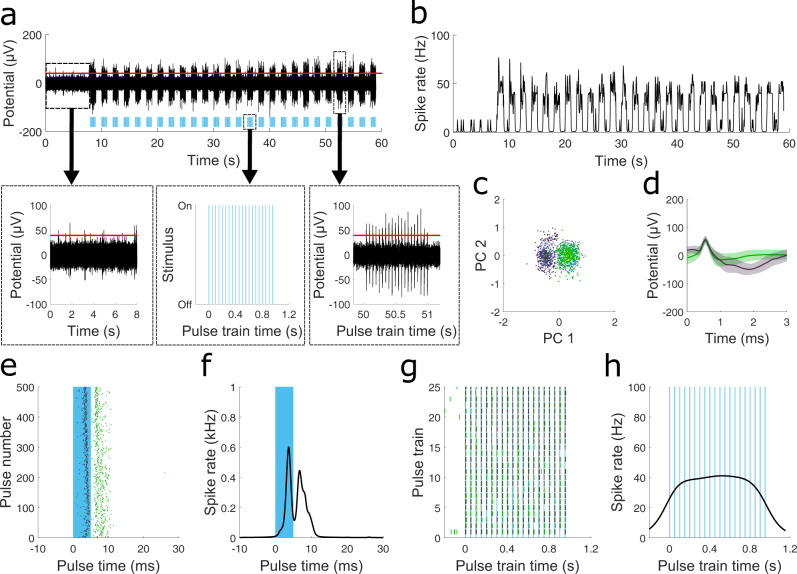
Concomitant optical stimulation and electrical recording in live Thy1-ChR2-YFP mice with the EO-Flex probes. **a** Optically evoked neural activity using a 20 Hz pulse train (blue bars) of 473 nm light (pulse width of 4.95 ms) at a tip power of 61 µW cycled on and off at 1 Hz. The threshold line (red) was set as defined^36^ in Fig. 2. **b** Spike rate plot for the recording in (**a**). **c** Principal component (PC) plot for the optically evoked neural activity. Separate clusters are colored differently. **d** Average waveforms (solid green or black line) for each cluster in (**c**) along with one standard deviation (shaded region). **e** Each cluster (color-coordinated black or green) plotted over time along with the window of a single pulse (blue bar). **f** Bayesian kernel smoothing estimate of spiking rate for each stimulation period. **g** Colored raster plot showing the occurrence of waveforms from (**d**). **h** Calculated average spike rate over the 1-s duration of a single pulsing cycle.

To demonstrate the EO-Flex probes’ ability to optically evoke neural activity while simultaneously electrically recording with the same probe, we performed experiments in anesthetized Thy1-ChR2-YFP mice with blue light-activated ion channel Channelrhodopsin-2 (ChR2) expression in neurons. Probes were inserted into cortical layer 2/3 under visual control. A 473 nm diode-pumped solid-state (DPSS) laser, suitable for exciting ChR2, was coupled into the probe, and stimulation parameters were swept systematically (Figs. S6, S8, S9). We varied the stimulation frequency (10–50 Hz), pulse width (0.6–9.8 ms), and output power (5–208 µW) to determine the optimal settings to excite ChR2-expressing neurons. Using waveform analysis on simultaneously recorded electrical activity, we found that a minimum power of 29 µW (2849 mW mm^−2^) was required for the firing of the cells in sync with the optical pulse train (Fig. S6). In the example recording shown in Fig. 4a–b, PCA combined with a mixed Gaussian fit for the clustering of the data yielded two primary clusters (Fig. 4c) with two different waveforms (Fig. 4d) occurring during the stimulation period (Fig. 4e–g). We found minimal interference (e.g., Becquerel effect) between the proximal optical and electrical channels, as demonstrated by testing the EO-Flex devices on non-transgenic animals at similar maximum optical power and stimulation frequencies (Fig. S10). Further validation of the absence of the Becquerel effect was shown by retracting the EO-Flex probe away from ChR2-expressing neurons, or placing it in a buffer solution, while optically pumping at similar laser power (208 µW) using the same optogenetic pulse trains (Fig. S11). At this maximum power, neural circuits responded with minimal temporal lag (Fig. S6f) and could follow frequency stimuli up to 40 Hz before struggling to maintain synced firing (Fig. S9).

The ability of EO-Flex probes to optically evoke neural activity was further verified by two-photon calcium imaging in Vglut2-GCaMP6f mice injected with an AAV2-CaMKII-C1V1-mCherry vector (see Methods). Four to five weeks after the cortical injection, EO-Flex probes were inserted into layer 2/3 regions expressing the green light-activated opsin C1V1. A 556 nm DPSS laser was coupled to the EO-Flex probe, and stimulation parameters were swept while simultaneously monitoring neuronal calcium transients. Delivered optical pulses led to correlated calcium spiking in C1V1-positive neurons within the field of view (Fig. S12). The successful optical evocation of neural activity was also verified by simultaneous electrical recordings (Fig. S12e). Together, our in vivo data demonstrate the ability of the EO-Flex probes to electrically record and optically modulate neural activity in the intact brain.

The EO-Flex probes allow targeting and entraining of opsin-expressing cells at firing rates ranging from 10 to 50 Hz (Fig. S9). While the minimum power (29 µW) to reliably activate neurons is higher than in previous reports (1–10 mW mm^−2^)^15,26,27^, it should be noted that due to the EO-Flex probes’ small optical core (3.6 µm), and anticipated light absorption and scattering from the tissue, higher intensities are expected to create illumination volumes that are large and strong enough to recruit neurons successfully compared to conventional larger core fiber optics. Monte Carlo simulations in Fig. S6b indicate that at a 29 µW stimulation power the optical power density drops below the optogenetic threshold of 1 mW mm^−2^ at around 1.2 mm from the tip. Even at the maximum stimulation power utilized in our optogenetic experiments (208 µW or 20,435 mW mm^−2^ at the probe tip), we did not observe any adverse cellular effects (e.g., sustained changes in firing rate or calcium levels) (Figs. S10–12). Recent studies have suggested that continuous optical exposure with powers <0.25 mW results in no temperature effects on neural activity (i.e., degraded electrical signals over the stimulation period)^28^. To further ensure minimal optical heating effects on the neural tissue, we utilized short pulse widths and optical powers of less than 250 µW (Figs. S6–10). Minimal heating effects are expected when using these illumination parameters, which was verified by applying previously validated heating models to the EO-Flex probes (Fig. S7b)^29^.

**Fig. 5:**
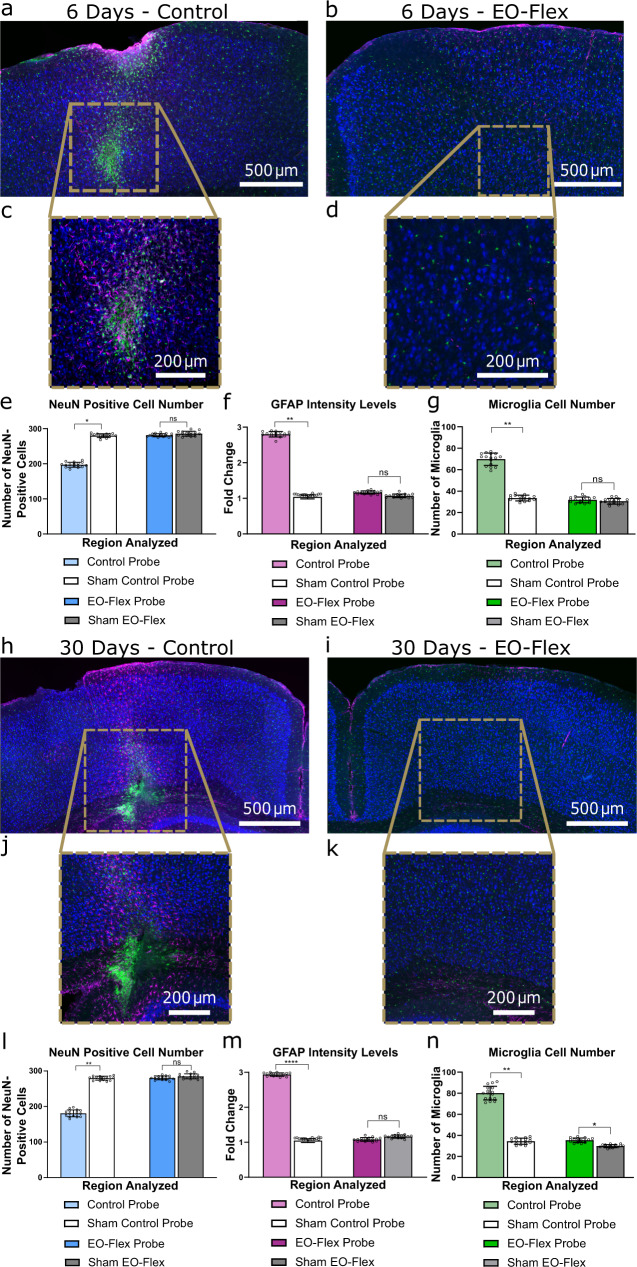
EO-Flex probes evoke minimal tissue responses compared to multimode fibers commonly used in optogenetic experiments. **a**, **b** Optical images showing 20 µm thick coronal brain sections around the multimode fiber (**a**) and EO-Flex probe (**b**) implantation sites. Both the multimode fiber (diameter, 250 µm) and EO-Flex probe (diameter, 12 µm) were advanced to an ~1 mm depth into the cortex. The images were taken 6 days after implantation in heterozygous Cx3cr1-GFP mice with labeled microglia (green). The sections were co-stained with anti-NeuN (blue) and anti-GFAP (magenta) antibodies to label neurons and astrocytes, respectively. **c**, **d** Higher resolution image of a zoomed-in region of (**a**, **b**) where the probe tips were located. **e**–**g** Population analysis (*n* = 16 sections from two animals) showing the impact of the multimode fiber or EO-Flex probe implantation on neuronal cell numbers (**e**), astrocyte reactivity as measured by GFAP expression level (**f**), and microglia reactivity as measured by microglial cell number (**g**) for the 6-day implantation. Cellular responses were quantified and averaged across two 150 µm × 1 mm analysis regions flanking each insertion site. To distinguish surgery from probe-related tissue responses, an additional craniotomy of comparable size was made 0.7 mm lateral to each device implantation site. The same analysis approach was used to quantify cellular responses at this sham surgery site. Two-tailed paired *t*-tests determined *P* values. **h**, **i** Coronal brain section optical images taken 30 days after multimode fiber (**h**) and EO-Flex probe (**i**) implantation. **j**, **k** Higher resolution image of a zoomed-in region of (**h**) and (**i**) where the probe tips were located. **l**–**n** Corresponding population analysis (*n* = 16 sections from two animals) showing neuronal cell numbers (**l**), astrocyte reactivity (**m**), and microglia reactivity (**n**) for the 30-day implantation. Two-tailed paired *t*-tests determined *P* values. The following convention was used to indicate *P* values: “ns” indicates *P* > 0.05, “*” indicates 0.01 < *P* ≤ 0.05, “**” indicates 0.001 < *P* ≤ 0.01, and “***” indicates 0.0001 < *P* ≤ 0.001. All bar plots are presented as mean ± s.e.m.

Next, we evaluated the brain’s response to probe implantation. EO-Flex probes were implanted into the cortex of heterozygous Cx3cr1-GFP mice with labeled microglia for 6 and 30 days. A 250 µm-diameter multimode fiber, commonly used in optogenetic experiments, was inserted using the same stereotaxic coordinates but on the opposite hemisphere for comparison. Serial brain sections were prepared that included both implantation sites. Tissue slices were co-stained with anti-GFAP and anti-NeuN antibodies to quantify reactive astrogliosis and neuronal loss, respectively (*n* = 4 mice; *N* = 8 sections per mouse) (Fig. 5 and Figs. S14, 15). At day 6 after implantation, we found that the multimode fiber led to significant neuronal loss, a 2.08 ± 0.23-fold increase in microglia numbers, and a 2.68 ± 0.60-fold increase in GFAP levels (Fig. 5e–g). In contrast, the EO-Flex probes showed no significant decrease in NeuN-positive cells or increase in microglia numbers or GFAP levels around the insertion site (Fig. 5e–g). At day 30 after implantation, neuronal loss was again observed for the implanted control multimode fiber, as well as a 2.33 ± 0.27-fold increase in microglia numbers, and a 2.81 ± 0.63-fold increase in GFAP levels (Fig. 5l–n). In contrast, the EO-Flex probes showed no significant decrease in NeuN-positive cells or increase in GFAP levels, but a small increase in microglia numbers (Fig. 5l–n). These results indicate that tissue responses to EO-Flex probe insertion or movement during the implantation period are negligible at time points when inflammatory responses are typically most prominent, and considerably smaller compared to standard probes used for optogenetic experiments^30^. Finally, given this minimal immune response, we also performed chronic recordings up to 30 days after EO-Flex probe implantation. These recordings revealed excellent signal-to-noise ratios across all investigated time points (day 0, 1, 2, 6, and 30) (Fig. S13).

## Discussion

In summary, we report on the fabrication of multi-modal coaxial microprobes and demonstrate their ability to optically stimulate and electrically record from intrinsic neural circuits with minimal interference between the two modalities. The small footprint and high aspect ratio of the EO-Flex probes allow minimally invasive interfacing with neural circuits. Further size reduction is possible with this coaxial design using smaller fiber optic cores; however, the tradeoff is an increase in optical losses and electrical impedance (Fig. S3). Although the probes’ capabilities were only tested in the brain, as a platform with excellent control over probe diameter and length, the choice of cladding materials with various chemical compositions, inherent mechanical flexibility, and a clear route to scaling up probe densities (e.g., translating the cladding deposition process to fiber bundles) (Fig. S16c), this technology should find immediate applications as minimally invasive interfaces in diverse nervous system regions, including the spinal cord and peripheral nerves.

Developing probes that can reach even deeper brain regions is straightforward with the developed fabrication protocols as virtually any microfiber length can be generated (Fig. S16a). However, for a given set of cladding layers, the probe’s stiffness decreases with length. Therefore, longer probes might require additional tactics to overcome low buckling forces during the insertion process (e.g., dissolvable sugar coatings, or rigid polymer layers)^31^. Alternatively, a surgical incision in the dura could facilitate probe insertion. Regardless of probe length, our implantation studies demonstrated that the small-footprint EO-Flex probes have a drastically reduced immune response compared to standard multimode fibers.

## Methods

### Probe fabrication

Silica microfibers (core and total diameters: 3.63 ± 0.31 µm and 5.60 ± 0.42 µm, respectively) with lengths varying between 500 µm and 1 cm were generated from leeched fiber optic bundles (Schott, Part no. 1573179). After cleaving, individual fibers were dispersed onto a silicon substrate. A tungsten needle mounted on a three-axis micromanipulator was used to position the microfibers near a substrate edge with one end of the fiber being suspended >100 µm from the edge.

To enable efficient optical coupling of the waveguide to standard optogenetic hardware, a single-mode fiber (SMF) (Thorlabs S405-XP) with a mode field diameter (2.8–3.4 µm) slightly smaller than the microfiber core was chosen. To create a robust detachable interface for in vivo testing, the SMF was inserted into a ceramic ferrule (Thorlabs CF126-10) and secured in place with quick cure epoxy (DevCon #20445). The ferrule assemblies were then machine polished until a smooth coupling interface was observed through a fiber inspection scope (Thorlabs, FS200), and the opposing fiber end (for coupling to the waveguide) was cleaved using a ruby scribe (Thorlabs S90R). The ferrule assembly was mounted on a three-axis stage, and the scribed end was maneuvered into a droplet of UV-cured optical adhesive (Norland Optical Adhesive 81) until a small droplet formed at the end. Efficient coupling between SMF and micro-/nanofiber was achieved using active alignment under an upright optical microscope (Nikon; software: Amscope v3.7.9229.20170607) equipped with a 0.4 NA 20x objective after coupling a 544 nm He-Ne laser source into the SMF. After maximizing power coupling into the waveguide by translating the SMF, the NOA 81 adhesive was secured by exposing it to UV light (325 nm line from a HeCd laser) for a duration of 30 s while continuously moving the beam around the droplet.

Before depositing the metal layer, the probe assemblies were placed in a custom aluminum block holder to mask the bottom part of the ferrule where light is coupled into the assembly. This ensured that the optical coupling interface was masked during metallization. This block was placed on a rotating plate inside a sputtering chamber (Denton Discovery 18). A thin (<10 nm) adhesion layer of titanium (2.5 mTorr, 5 s, 200 W) was deposited, followed by a 300 nm thick layer of iridium oxide (IrO_x_) (12 mTorr, 15 min, 100 W, 5 sccm O_2_ flow) or 500 nm of platinum (Pt) (2.5 mTorr, 20 min, 200 W). Iridium oxide was chosen for its ~3x higher charge-injection capacity compared to conventional platinum layers, and its porous nature, which increases the electrochemical surface area of the metal layer^32^.

Together, these procedures yielded ferrule assemblies for repeatable mounting to an in vivo imaging and optogenetics setup (Fig. 2). Alternative interface designs (e.g., for probe arrays) are shown in Fig. S16.

### PEDOT-PSS deposition

To further lower the electrical impedance of the probes, a poly(3,4-ethylenedioxythiophene)-polystyrene sulfonate (PEDOT:PSS) layer was deposited on the IrO_x_. Probes were submersed (~100 µm of the probe tip) into a 0.01 M solution of EDOT (97%, Millipore-Sigma) with 2.5 mg/mL of poly(sodium styrene sulfonate) (PSS; Millipore-Sigma). The electrochemical deposition was performed using a platinum wire counter electrode and an Ag/AgCl reference electrode (CHI 111 P, CH Instruments) connected to an electrochemical potentiostat (VersaSTAT4) operating in the galvanostatic mode set to run at a current of 200 nA for 5–30 s. This yielded a 362 ± 137 nm thick polymer layer (Fig. 1e and Fig. S2). The PEDOT-IrO_x_ (or PEDOT-Pt) coated microfibers were then passivated with 1.5–2 µm of parylene-C using chemical vapor deposition (SCS Labcoater Deposition System; Specialty Coating Systems).

### In vitro probe characterization

A focused ion beam (FEI Scios Dual-beam) set to 5 nA at 30 kV was used to cleave off the end of the probe and expose the electrical and optical channels, revealing a final probe diameter of 8–12 µm for the microfiber cores. Electrochemical impedance spectroscopy (EIS) was carried out with the Versastat4 (running VersaStudio v.2.60.6) to determine probe impedance in a 1× phosphate-buffered saline (PBS) using the same reference and counter electrodes described above. Optical coupling efficiency was determined by measuring light output from a fiber optic patch cable (Thorlabs, P1405B-FC-5) using three light sources (473, 543, and 673 nm) interchangeably coupled into the cable. Light power was measured by placing the ferrule 5–10 mm away from the detector head of a digital power meter (Thorlabs, PM100D). A ceramic ferrule sleeve (Thorlabs, ADAL1) was then slid halfway onto the patch cable, and different EO-Flex probes were slid into the opposite end to couple light through. Light power from the tip of the EO-Flex probes was measured using a similar protocol to the patch cable.

### Animal subjects

All live animal procedures were performed following the guidelines of the National Institutes of Health (NIH) and were approved by the Institutional Animal Care and Use Committee (IACUC) at the Salk Institute under protocol number 13-00022. For combined optogenetic and electrophysiological experiments, we used *Thy1-ChR2-YFP* male mice (stock #007612; Jackson Laboratories; age: 10 months); for combined calcium imaging, optogenetics, and electrophysiological experiments, we used AAV2-CaMKII-C1V1-mCherry-injected *Vglut2-GCaMP6f* male mice (a custom cross between *Vglut2-Cre* knock-in and *Ai95D* mice; stock #028863 and #024105, respectively; Jackson Laboratories; age: 3 months); for immune response and all other studies, we used heterozygous *Cx3cr1-GFP* male mice (stock #005582; Jackson Laboratories; age: 9 weeks).

### Stereotactic viral vector injection

Surgical procedures closely followed previously established protocols^33,34^. Briefly, thin-wall glass pipettes were pulled on a Sutter Flaming/Brown micropipette puller (model P-97). Pipette tips were cut at an acute angle under 10× magnification using sterile techniques. Tip diameters were typically 15–20 μm. Pipettes that did not result in sharp bevels nor had larger tip diameters were discarded. Millimeter tick marks were made on each pulled needle to measure the virus volume injected into the brain.

Mice were anesthetized with isoflurane (4% for induction; 1–1.5% for maintenance) and positioned in a computer-assisted stereotactic system with digital coordinate readout and atlas targeting (Angle Two, Leica). Body temperature was maintained at 36–37 °C with a DC temperature controller, and ophthalmic ointment was used to prevent the eyes from drying. A small amount of depilator cream (Nair) was used to remove hair at the designated skin incision site. The skin was cleaned and sterilized with a two-stage scrub of betadine and 70% ethanol. A midline incision was made beginning just posterior to the eyes and ending just passed the lambda suture. The scalp was pulled open, and the periosteum was cleaned using a scalpel and forceps to expose the desired hemisphere for calibrating the digital atlas and coordinate marking. Once reference points (bregma and lambda) were positioned using the pipette tip and entered into the program, the desired target was set on the digital atlas. The injection pipette was carefully moved to the target site (coordinates: AP −1.5 mm, ML 1.5 mm). Next, the craniotomy site was marked, and an electrical micro-drill with a fluted bit (0.5 mm tip diameter) was used to thin a 0.5–1 mm diameter part of the bone over the target injection site. Once the bone was thin enough to flex gently, a sterile 30 G needle with an attached syringe was used to carefully cut and lift a small (0.3–0.4 mm) segment of bone.

For injection, a drop of the virus was carefully pipetted onto parafilm (1–2 μl) for filling the pulled injection needle with the desired volume. Once loaded with sufficient volume, the injection needle was slowly lowered into the brain until the target depth (DV 0.2 mm) was reached. Manual pressure was applied using a 30-mL syringe connected by shrink tubing, and 0.4 μl of the AAV2-CaMKII-C1V1-mCherry vector (6.1E + 12 VP/mL; undiluted; UNC Vector Core) was slowly injected over 5–10 min. Once the virus was injected, the syringe’s pressure valve was locked. The position was maintained for ~10 min to allow the virus to spread and to avoid backflow upon needle retraction. Following the injection, the skin was sutured along the incision. Mice were given subcutaneous Buprenex SR (0.5 mg per kg) and allowed to recover before placement in their home cage. The vector was allowed to express for 4–5 weeks before in vivo recordings.

### Animal preparation for in vivo recordings

Surgical procedures closely followed established protocols^23,35^. Briefly, mice were anesthetized with isoflurane (4–5% for induction; 1–1.5% for maintenance) and implanted with a head plate on a custom surgical bed (Thorlabs). Body temperature was maintained at 36–37 °C with a DC temperature control system and ophthalmic ointment was used to prevent the eyes from drying. Depilator cream (Nair) was used to remove hair at the designated skin incision site. The skin was thoroughly cleansed and disinfected with a two-stage scrub of betadine and 70% ethanol. A scalp portion was surgically removed to expose frontal, parietal, and interparietal skull segments. Scalp edges were attached to the skull’s lateral sides using a tissue-compatible adhesive (Vetbond; 3 M). A custom-machined metal plate was affixed to the skull with dental cement (cat. #H00335; Coltene Whaledent), allowing the head to be stabilized with a custom holder. Mice were given Buprenex SR (0.5 mg/kg) before relief from anesthesia and allowed to recover for at least three days before further preparation.

For combined imaging and electrophysiological recordings, an ~2 mm × 4 mm diameter craniotomy was performed over the target area (e.g., the AAV vector injection site). The dura mater overlying the cortex was kept intact. Tissue motion was controlled by covering the exposed tissue with a 1% agarose solution and coverslip. The coverslip was affixed to the skull with Vetbond (3 M) and dental cement. To enable probe entry into the cortex, the agarose and coverslip were cut on one side to be flush with the craniotomy. Recordings started immediately after optical window preparation. The depth of anesthesia was monitored throughout the experiment and adjusted as needed to maintain a breath rate of approximately 55–65 breaths per minute. Saline was supplemented as needed to compensate for fluid loss.

For electro-optical measurements under awake conditions, mice were first habituated to head restraint on a spherical treadmill (typically three sessions, 30–90 min/session, one session/day on three consecutive days). Following habituation, an ~0.3–0.5 mm diameter craniotomy was performed over the target area (barrel cortex; coordinates: AP −1.0 mm, ML 3.0 mm) under general anesthesia. Mice were then transferred to the spherical treadmill and allowed to recover from anesthesia for at least 30–60 min, depending on the duration they had spent under anesthesia. Following electro-optical measurements, the probe was locked into position by first covering the probe/tissue interface with 1% agarose solution and then applying Vetbond (3 M) and dental cement, thereby affixing the ferrule to the skull. Mice were allowed to recover in their home cage before subsequent recordings on different days.

### In vivo electrophysiology

To characterize the EO-Flex probes’ electrophysiological properties in vivo, we performed extracellular single- and multi-unit recordings in the cortex of isoflurane-anesthetized and awake mice, similar to our previous work^23,24^. The EO-Flex probes’ electrical channel was connected to the positive terminal of a high impedance head stage (Model 1800 microelectrode AC amplifier, A-M Systems) using a custom adapter, whereas the negative terminal and ground was connected to an Ag/AgCl wire inserted above the cerebellar cortex. The adapter consisted of a ceramic block with an embedded patch cable end and removable copper clip soldered to a single core head stage wire. EO-Flex probes were mated with this adapter by sliding a ferrule sleeve onto the patch cable end, sliding the probe into this assembly, and then lowering the copper clip to contact the metal layer on the EO-Flex ferrule.

To allow targeted tissue insertion and precise positioning of the probe, the adapter was mounted to a motorized micromanipulator (MP-225, Sutter Instrument Company) oriented at a defined angle with respect to the skull (e.g., ~60 degrees for combined imaging and electrophysiology, and ~0 degrees for measurements without imaging). After positioning the tip of the EO-Flex probe near the edge of the craniotomy, a few drops of physiological saline were pipetted onto the skull opening to facilitate mechanical insertion through the tissue interface (Fig. 2a).

Precise positioning was aided by passing the differential amplifier’s output through a speaker to serve as auditory feedback for probe proximity to active cells. The raw electrode signal was amplified, filtered (low cut-off, 300 Hz; high cut-off, 5 kHz; gain, 1000×), digitized (20 kHz; using DAQExpress 2.0), and stored on disk for off-line analysis. Positioning of the probe’s tip near neuronal cell bodies was further aided by visual feedback in experiments involving imaging in fluorescent indicator-expressing transgenic mice.

Electrical stimulation (Fig. S5) involved EO-Flex probe-mediated current pulse delivery (0 to 300 µA amplitude, 100 Hz stimulation frequency, 0.2 ms pulse width, 1 Hz stimulation period) with an isolated pulse stimulator (Model 2100; A-M Systems) connected to a function generator.

### Whisker stimulation

The barrel cortex in a given hemisphere receives sensory input from whiskers located on the opposite side of the body. To deflect whiskers contralateral to the probe’s recording location, we delivered air puffs with a micropipette connected through plastic tubing to a function generator-controlled pressure system (Picospritzer III; Parker Hannifin Co.). The function generator also operated an infrared LED positioned outside the animal’s but within the video camera’s field of view for synchronizing the analog and video data. The micropipette was connected to a motorized micromanipulator (MP-225, Sutter Instrument Company), allowing precise control over the whiskers being stimulated. Air pressure was directed away from the skin and eye and delivered in rostra-caudal direction. Air puff stimuli consisted of 2 s “on” followed by 2 s “off”, with varying pulse frequencies (2–5 Hz) and widths (20–100 ms).

### Two-photon microscopy

In vivo imaging was performed^23,33^ by utilizing a Movable Objective Microscope (Sutter Instrument Company) equipped with a pulsed femtosecond Ti:Sapphire laser (Chameleon Ultra II, Coherent), two fluorescence detection channels (emission filters: ET525/70 M and ET605/70 M (Chroma); dichroic beam splitter: 565DCXR (Chroma); photomultiplier tubes: H7422-40 GaAsP (Hamamatsu)), and MPScope image acquisition software (Kleinfeld lab, UCSD). The laser excitation wavelength was set to 920 nm. The average laser power was <10–15 mW at the tissue surface and adjusted with depth to compensate for signal loss due to scattering and absorption. A 16× 0.8 NA (CFI75, Nikon) or 40× 0.8 NA (LUMPLFLN, Olympus) water-immersion objective was used for light delivery and collection. Spontaneous and optically evoked calcium activity was recorded in optical planes near the probe tip (frame rate, 8.14 Hz). To minimize the Becquerel effect mediated artifacts in electrical recordings, the imaging laser power was kept to a minimum. To record optically evoked calcium transients in optogenetic experiments, we synchronized the image frame rate with optical pulse train delivery and adjusted the phase such that regions in front of the probe tip were scanned when the DPSS laser was off (Fig. S12).

### In vivo optogenetics

To excite ChR2 or C1V1, respectively, the light from a 200-mW 473 or 556 nm DPSS laser (CNI), directly modulated by an external function generator signal, was coupled into the probe. Light coupling into the probe was achieved by sliding the polished end of the ferrule into a ceramic sleeve and then sliding it onto the end of a custom fiber patch cable. Each stimulation trial lasted around 60 s, with the initial 5–10 s designated for recording spontaneous activity before the optical pulse train was delivered (stimulation power, 6–208 µW; pulse width, 0.6–9.8 ms; stimulation frequency, 10–50 Hz; duration, 1 s; inter-stimulus-interval, 1 s between pulse trains).

### Tissue response assessment

Heterozygous Cx3cr1-GFP mice (male, 9 weeks old) with labeled microglia were implanted with an EO-Flex probe and a 250 μm-diameter multimode fiber suited for optogenetic deep brain stimulation on opposite hemispheres (±1.45 mm from midline). For implantation, an electrical micro-drill with a fluted bit (0.5 mm tip diameter) was used to thin a 0.5–1 mm diameter part of the bone. Once the bone was thin enough to flex gently, a sterile 30 G needle with an attached syringe was used to carefully cut and lift a small (0.3–0.4 mm) segment of bone. The probe or multimode fiber was advanced through this opening under visual control to a depth of approximately 1 mm using a computer-assisted stereotactic system (Angle Two, Leica). Dental cement was used to secure the devices in place. The firm bonding of the dental cement to the skull was facilitated by scarifying it with a bone scraper (Fine Science Tools). To distinguish surgery from probe related tissue responses, we performed additional craniotomies 0.7 mm lateral from the device implantation sites (Figs. S14, S15). To assess tissue inflammatory responses, mice were sacrificed 6 or 30 days after device implantation using CO_2_ asphyxiation following IACUC guidelines. Transcardial perfusion was performed with 10% sucrose in PBS, followed by freshly prepared 4% PFA in PBS. Both hemispheres were post-fixed in 4% PFA in PBS overnight and subsequently infiltrated in 30% sucrose in PBS for one day and flash frozen in a TBS tissue freezing medium. The implanted hemispheres were coronally cryo-sectioned at 20 μm, air-dried overnight, and subsequently processed for staining. Sections were incubated overnight at 4 °C with primary antibody diluted in blocking buffer, then washed in PBS 0.1% Tween-20, and incubated for two hours at 22–24 °C in the dark with fluorophore-coupled secondary antibodies. Sections were washed, sealed with Prolong Gold Antifade Mountant (Thermo Fisher Scientific), and stored at 4 °C. Primary antibodies included anti-GFAP (mouse monoclonal; EMD Millipore; cat. #MAB3402; RRID: AB_94844; 1:250 dilution) and anti-NeuN (rabbit polyclonal; EMD Millipore; cat. #ABN78; RRID: AB_10807945; 1:100 dilution). Secondary antibodies (1:100) included Alexa Fluor 405 goat anti-rabbit (Thermo Fisher Scientific; cat. #A-31556; RRID: AB_221605) and Alexa Fluor 633 goat anti-mouse (Thermo Fisher Scientific; cat. #A-21052; RRID: AB_2535719). Confocal imaging of stained tissue sections was performed on a Zeiss LSM 710 (software: ZEN Black, Zeiss v2011). Three-channel tiled z-stacks were acquired to produce images of whole-tissue sections (Fig. 5 and Figs. S14, S15). Image size was 1024 × 1024 pixels stitched into 3–5 × 3–5 tiles. Images were taken with an Olympus 20 × 0.8 NA air-matched objective.

### Data processing and statistical analyses

Neural activity was considered a spike if its amplitude crossed a threshold determined by ${{{{{\rm{Threshold}}}}}}=4* {{{{{\rm{median}}}}}}\left(\frac{{{{{{\rm{|Recording|}}}}}}}{0.675}\right)$ ^36^. All observed spikes were then sorted according to the first two principal components into clusters using a mixed Gaussian fitting with the number of clusters optimized according to the Calinksi–Harabasz metric for cluster analysis^37^ calculated in MATLAB (R2019b). Average firing rates were calculated using the Bayesian Adaptive Kernel Smoother (BAKS) (v2017; https://github.com/nurahmadi/BAKS). Monte Carlo simulations were used to determine the propagation and illumination volume of the EO-Flex probe at different powers (Fig. S6b).

Optogenetic heating profiles were created using previous models^29^ utilizing the Pennes bio-heat equation. Simulation parameters were for an EO-Flex probe optical radius of 1.8 µm, a wavelength of 470 nm, power of 1 mW, or 208 µW, and a cylindrical radius of 10 µm for temperature averaging in the time-based simulations (Fig. S7).

Peristimulus plots correlating optical stimuli with spiking events were calculated using kernel bandwidth optimization, which has been shown to accurately estimate the underlying spiking rate (Fig. 4b)^25^.

Analysis of the two-photon calcium imaging data was performed using Suite2p (Fig. S12)^38^. Optically evoked calcium spiking was observed in optical planes near the probe tip. Our analysis focused on the optical planes in which at least three cellular-size regions of interest (ROIs) consistently responded throughout the stimulation period.

Immunostaining data were processed, analyzed, and plotted using ImageJ (v1.53f51), Imaris (v9.2; Oxford Instruments), and Prism (v8.4.3; GraphPad Prism) software. All data were represented as mean ± s.e.m. Group sample sizes were chosen based on previous studies and power analysis. Two-tailed paired *t*-tests determined *P* values. The following convention was used to indicate *P* values: “ns” indicates *P* > 0.05, “*” indicates 0.01 < *P* ≤ 0.05, “**” indicates 0.001 < *P* ≤ 0.01, and “***” indicates 0.0001 < *P* ≤ 0.001.

### Reporting summary

Further information on research design is available in the Nature Research Reporting Summary linked to this article.

## Supplementary information

Supplementary Information

Supplementary Video 1

Supplementary Video 2

Reporting Summary

## Data Availability

Source data are provided with this paper. Additional data that support the findings of this study are available from the corresponding authors upon request. Source data are provided with this paper.

## Source data

Source Data

## Code availability

The custom code used to process the data and create the figures is available on GitHub (https://github.com/Spencer-W/EO-Flex-Algorithms.git). It will also be available from the corresponding authors upon request.
