# Supplementary material for: Electro-optical mechanically flexible coaxial microprobes for minimally invasive interfacing with intrinsic neural circuits
Source: Nat Commun. 2022 Jun 7;13:3286. doi: 10.1038/s41467-022-30275-x (PMC9174211; doi:10.1038/s41467-022-30275-x)
Supplement: Supplementary file 1 — Supplementary Information [file 41467_2022_30275_MOESM1_ESM.pdf]

Supporting Information

Electro-optical mechanically flexible coaxial microprobes for minimally invasive interfacing with intrinsic neural circuits

Spencer Ward[1], Conor Riley[2], Erin M. Carey[3], Jenny Nguyen[2], Sadik Esener[1,2], Axel Nimmerjahn[3*], and Donald J. Sirbuly[2,4*]

[1]Department of Electrical and Computer Engineering, [2]Department of Nanoengineering, University of California, San Diego, La Jolla, CA 92093, USA. [3]Waitt Advanced Biophotonics Center, Salk Institute for Biological Studies, La Jolla, CA 92037, USA. [4]Materials Science and Engineering, University of California, San Diego, La Jolla, CA 92093, USA.

*E-mail: animmerj@salk.edu; dsirbuly@eng.ucsd.edu

Methods

Probe fabrication. EO-Flex probes were fabricated using one of two waveguides as the optical core: a) silica microfibers (SiO_x) (Fig. 1 and Fig. S2), or b) single crystalline tin dioxide (SnO_2) nanofibers (Fig. S3).

The SnO_2 nanofibers were synthesized using thermal evaporation of SnO powders at high temperatures according to published protocols.[1] Ceramic combustion boats were loaded with 1-5 grams of tin monoxide powder and placed in a tube furnace. The system was pumped down to < 1 mTorr as the furnace was turned on to 1000 °C. At operating temperature, system pressures were typically around 300 mTorr. The system was allowed to run for an hour, after which the furnace was turned off, and the system was allowed to cool while the vacuum pump remained on. The combustion boat was then removed, and nanowires found on the boat's rim

were transferred to a silicon substrate to facilitate coupling to a cleaved SMF (Fig. 1, S3; main text).

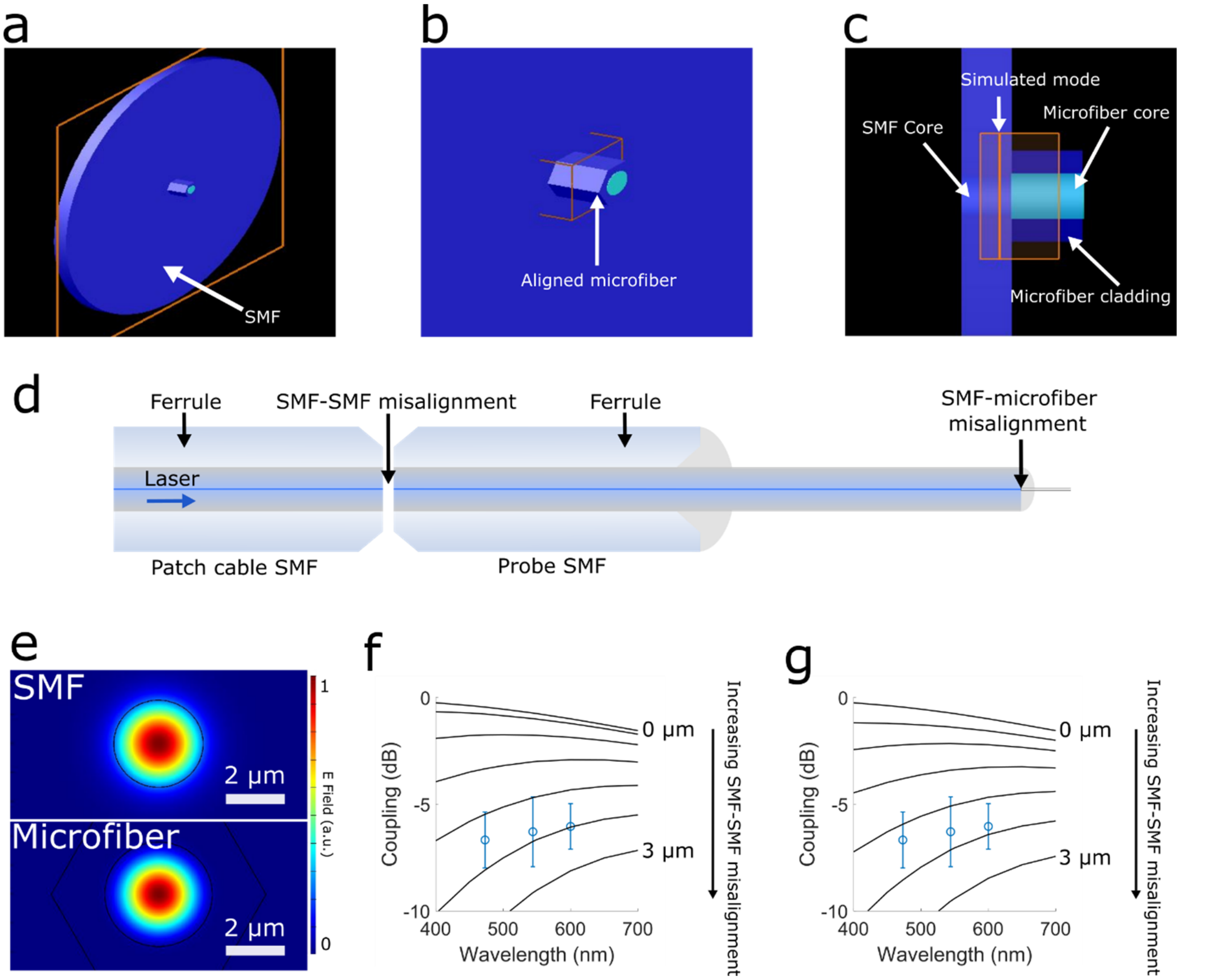

Figure S1. Finite element modelling of EM modes (Lumerical MODE) to investigate the theoretical optical coupling between the single-mode fiber (SMF) and microfiber. (a) Simulation geometry of a SMF fiber that has its core perfectly aligned with the core of the microfiber. (b) Zoom-in of the SMF-microfiber interface showing a 10 nm mesh used to quantify the coupling efficiency. (c) Side profile of (b) with the design and labels of the model. (d) Schematic of the optical coupling into the probe from the laser source showing where the mode misalignment was simulated (SMF-SMF and SMF-microfiber). (e) Mode profile simulated at 473 nm for both the SMF and the microfiber. (f) Simulated coupling efficiency assuming no misalignment between the SMF-microfiber interface and misalignment of the SMF-SMF interface from 0 to 3 μm (solid lines indicate every 0.5 μm of misalignment). The average coupling of 4 probes measured at 473 nm, 543 nm, and 600 nm is overlaid on simulated curves. The data are presented as the mean and standard deviation. (g) Calculated coupling efficiency assuming the SMF-microfiber interface has 500 nm of misalignment and similar misalignment of the SMF-

SMF interface as in (f). The same measured data set in (f) is overlaid on the new coupling curves. The data are presented as the mean and standard deviation. A comparison of simulation and measurements shows that optical coupling losses are mostly due to SMF-SMF misalignment (measured losses fall between 2 - 2.5 μm of SMF-SMF misalignment).

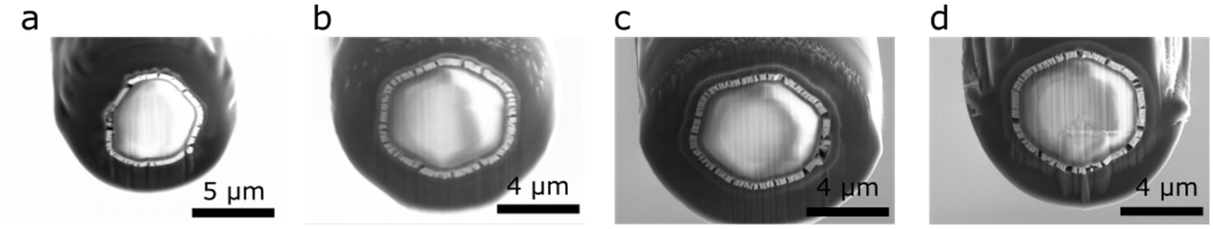

e

Device	Microfiber Diameter (μm)	Iridium Oxide Thickness (nm)	PEDOT-PSS Thickness (nm)	Parylene-C Thickness (μm)
Probe a	3.19	382	230	1.76
Probe b	3.74	437	259	1.73
Probe c	3.72	356	495	1.96
Probe d	3.89	338	466	1.57

Figure S2. (a-d) Electron micrographs of four microfiber EO-Flex probes with different PEDOT:PSS deposition conditions. Data was used to optimize the polymer thickness and quantify the dimensions of the other cladding layers. Micrographs for each probe and PEDOT deposition thickness were taken once. (e) Table of the different thicknesses for each layer deposited on the probes.

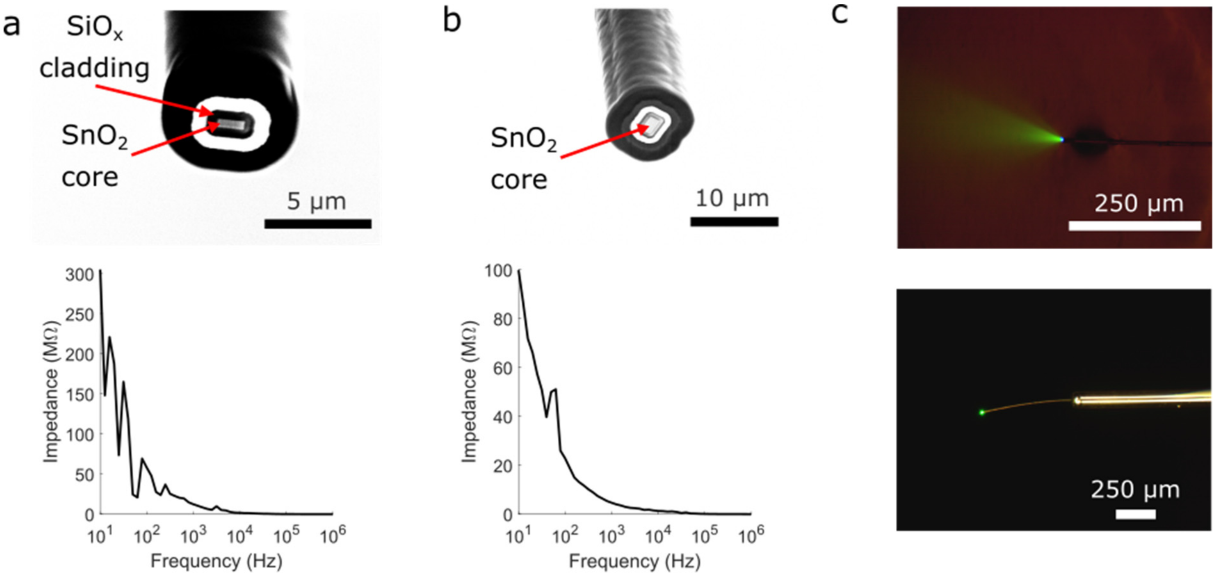

Figure S3. EO-Flex probes fabricated with a single crystalline tin dioxide (SnO_2) nanofiber waveguide as the optical core. (a) (top) Electron micrograph of a SnO_2 EO-Flex probe fabricated without the PEDOT-PSS layer. (bottom) EIS data of the probe showing an impedance of >20 MΩ at 1 kHz. (b) (top) Electron micrograph of a SnO_2 EO-Flex probe fabricated with the PEDOT-PSS layer. (bottom) EIS data of the probe showing a significant reduction in the impedance down to 5 MΩ at 1 kHz. (c) (top) The optical output of a SnO_2 EO-Flex probe in a fluorescent dye solution showing the exiting cone angle. (bottom) Optical image of the freestanding probe showing no light scattering at the SnO_2-SMF interface after cladding deposition. Micrographs were taken once for the tin dioxide probes shown above.

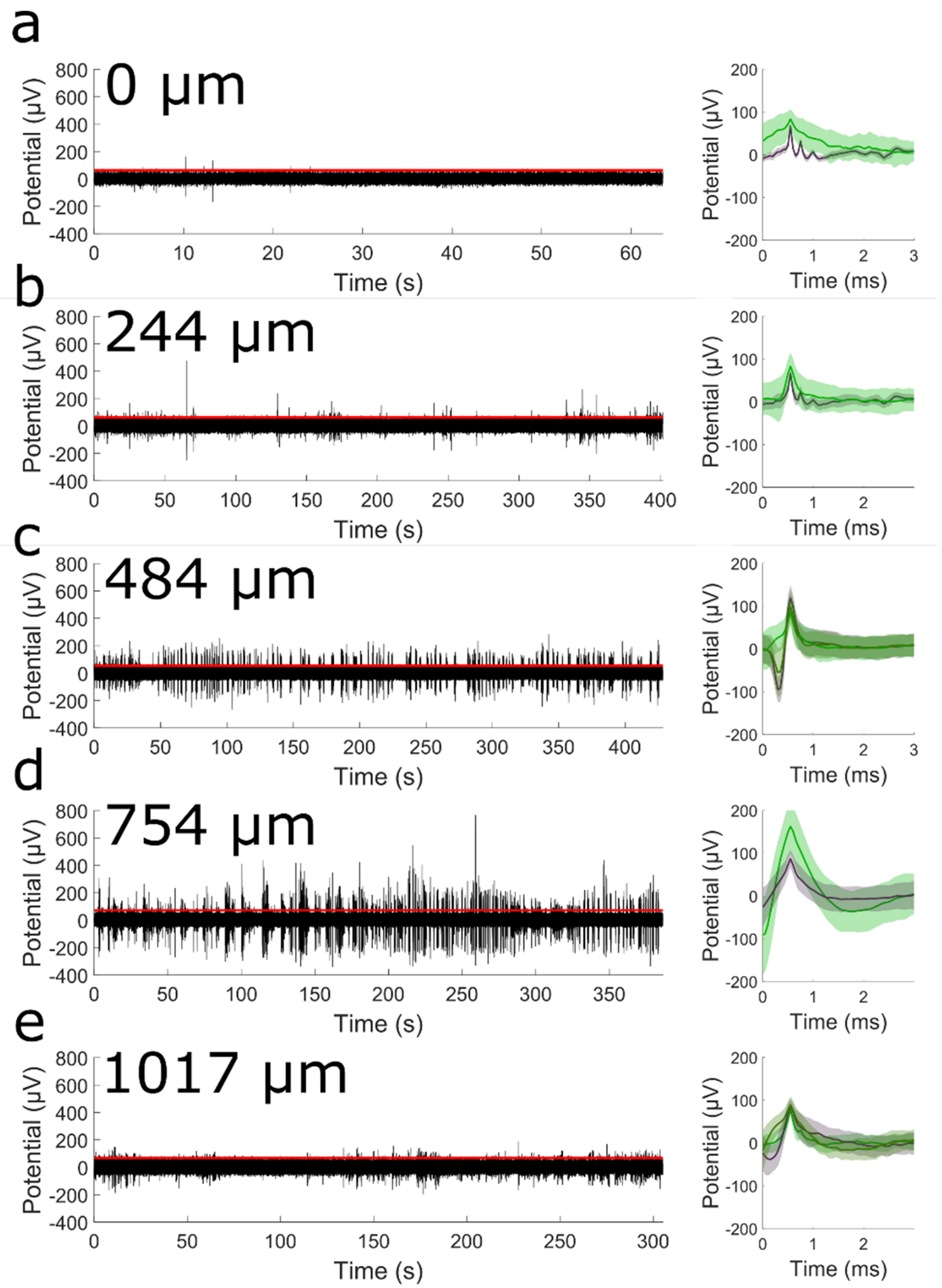

Figure S4. EO-Flex recordings across cortical layers of an anesthetized mouse. Insertion depths calculated from stereotactic coordinates are displayed with corresponding recordings. (a-e) Example recordings showing spontaneous activity at different probe insertion depths. Corresponding spike sorted average waveforms with one standard deviation (shaded) are shown on the right. Reduced spike amplitude and activity at 1017 µm insertion depth suggests entrance into the white matter beneath the cortex.

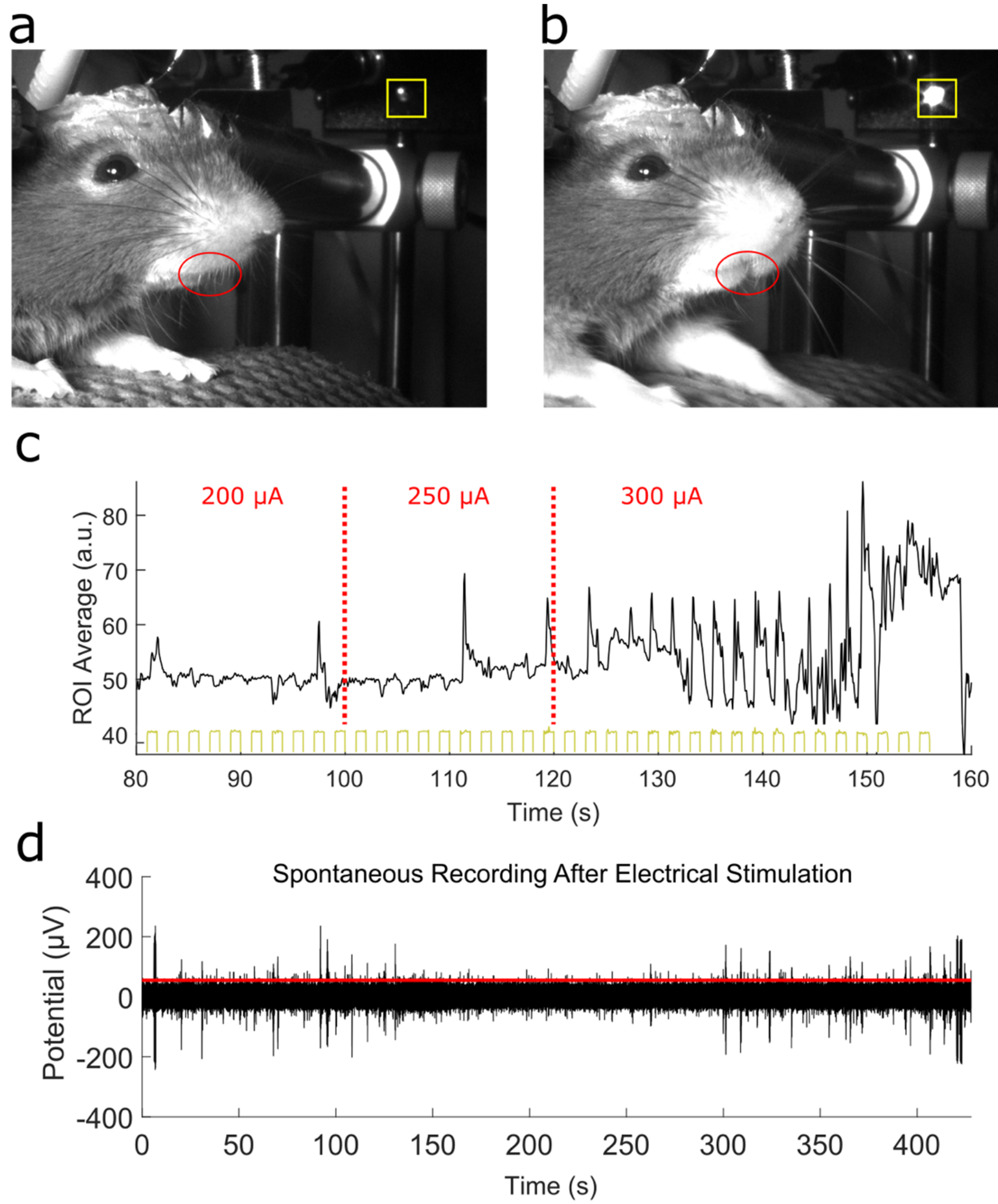

Figure S5. Electrical stimulation evoked whisker deflection with EO-Flex probes. In this example from an awake head-restrained mouse on a spherical treadmill, the probe was implanted into the barrel cortex for 30 days with its tip inserted to a depth of 761 μm. Stimulation current was ramped up from 0 μA to 300 μA while using a stimulation frequency of 100 Hz, pulse width of 0.2 ms, and 1 Hz stimulation period. (a) Video frame showing the resting animal before electrical stimulation. Electrical pulse train delivery was controlled by a function generator. This device also controlled an infrared LED (yellow box) for analog and video data synchronization. The red ROI indicates an analysis region around a group of whiskers. (b) Video frame showing whisker pad deflection during stimulus delivery. (c) Average pixel intensity (black) in the indicated ROI (red; panel a-b) as the current is increased in 50 μA steps every 20 s. The timing of stimulus delivery is indicated at the bottom (yellow trace). (d) Spontaneous recording acquired after the electrical stimulation demonstrating that the EO-Flex probe remained intact. Spikes were detected when the potential crosses the threshold (red line).

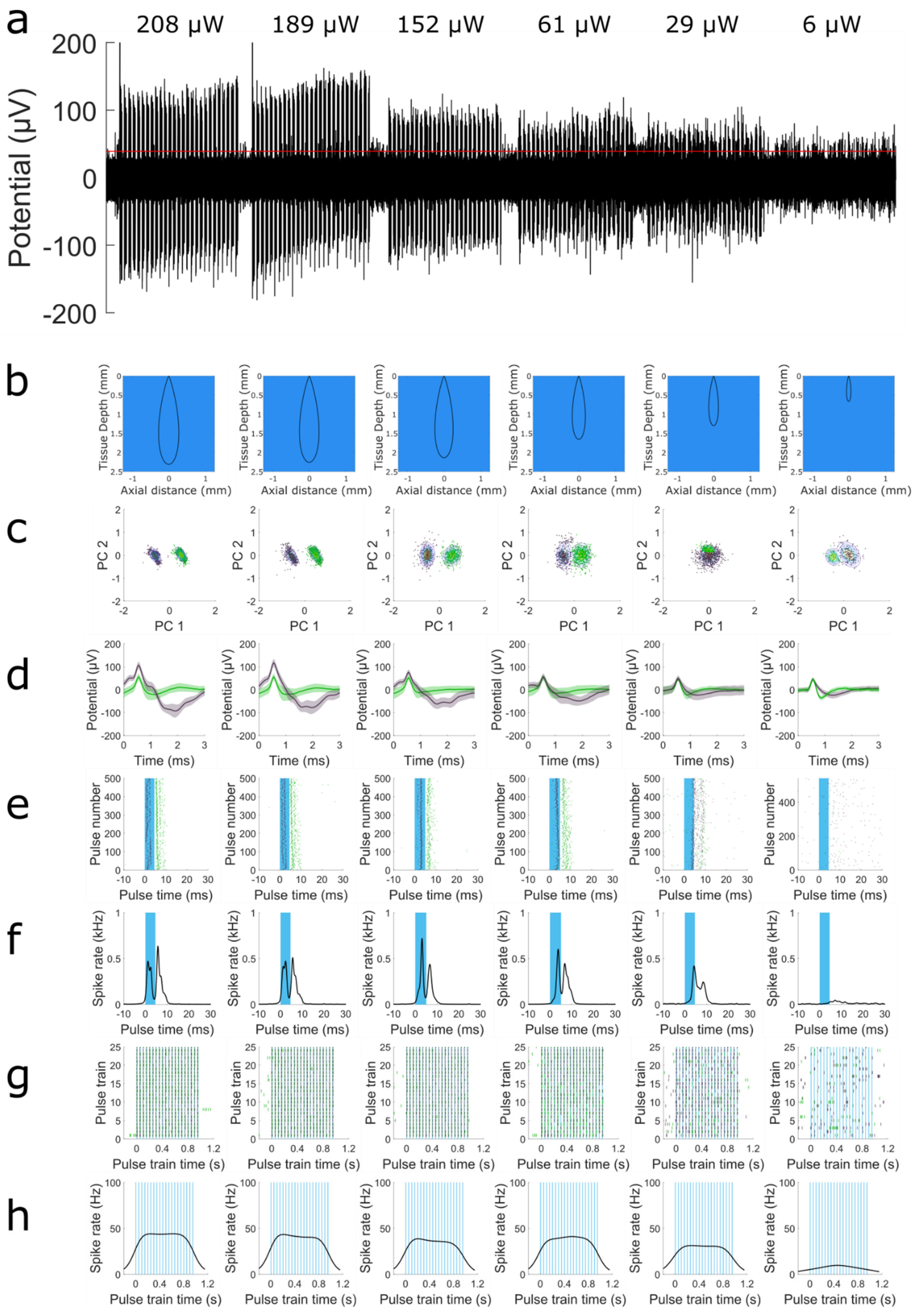

a
208 μW
189 μW
152 μW
61 μW
29 μW
6 μW
Potential (μV)
b
Tissue Depth (mm)
Axial distance (mm)
c
PC 2
PC 1
d
Potential (μV)
Time (ms)
e
Pulse number
Pulse time (ms)
f
Spike rate (kHz)
Pulse time (ms)
g
Pulse train
Pulse train time (s)
h
Spike rate (Hz)
Pulse train time (s)

Figure S6. EO-Flex testing in layer 2/3 of a live Thy1-ChR2-YFP mouse with light-activated protein expression in neurons as a function of optical stimulation power. All other stimulation parameters were held constant (pulse width, 4.5 ms; stimulation frequency, 20 Hz; on/off cycling, 1 Hz). The recording depth was ~250 μm. (a) Optically evoked neural activity using EO-Flex output powers of 208 μW (20,435 mW mm^{-2}), 189 μW (18568 mW mm^{-2}), 152 μW (14933 mW mm^{-2}), 61 μW (5,993 mW mm^{-2}), 29 μW (2,849 mW mm^{-2}), 6 μW (560 mW mm^{-2}). (b) Monte Carlo simulations of the scattering and absorption in neural tissue for each of the power values in (a). The solid line indicates where irradiance has fallen to 1 mW mm^{-2}. The simulations show that a light intensity of 208 μW could propagate up to 2.4 mm from the probe tip before the irradiance drops below 1 mW mm^{-2}. Simulation parameters were taken from recent studies[3], which estimated scattering and absorption coefficients of 0.125 mm^{-1} and 7.37 mm^{-1}, respectively. (c) The first two principal components (PCs) of respective electrical recordings plotted with the Calinski-Harabasz metric for determination of the number of clusters for mixed Gaussian fitting. (d) The average waveform for each cluster (solid line) from (c) with the shaded region representing one standard deviation. (e) Peri-stimulus plots for all optical pulses with spikes color-coordinated with the cluster from which they come. (f) Bayesian adaptive kernel smoother (BAKS) estimation for the firing rate over the time window around the optical pulses. (g) Peri-stimulus plot for each optical pulse train from high to low power. (h) BAKS estimation for the firing rate over the pulse train window.

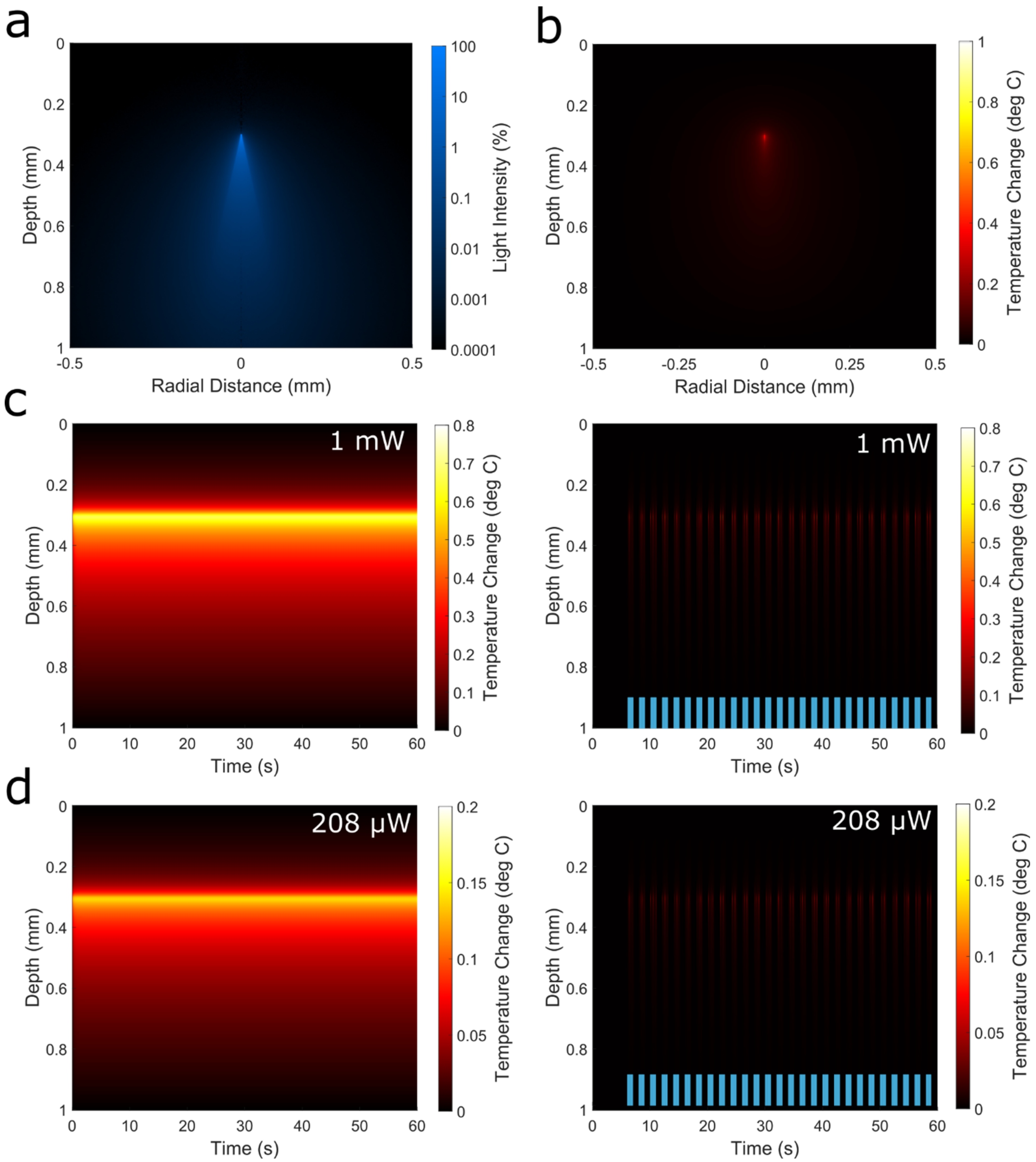

a
b
c
d
Depth (mm)
Radial Distance (mm)
Light Intensity (%)
Temperature Change (deg C)
Time (s)
1 mW
208 μW

Figure S7. Estimated light intensity distribution and heating around the EO-Flex probe tip for continuous or pulsed 470 nm EO-Flex light of 208 μW or 1 mW. For time-based simulations (c-d), the average temperature change was calculated within a cylinder of 20 μm radius around the probe with the probe tip located at a 0.3 mm depth. The results were obtained using previously validated optogenetic heating simulation software[4]. (a) Light output distribution for an EO-Flex probe whose tip is located at a 0.3 mm depth within a 1 x 1 mm simulation volume. (b) Heating profile around the EO-Flex probe tip pumped with continuous 470 nm light at 208 μW for 60s (maximum temperature 0.34° C). (c) Average temperature around the EO-Flex probe as a function of time for continuous (left, max 0.66 °C) and 20 Hz pulsed with 1 Hz on/off cycling (right, max 0.37 °C) of 470 nm light at a power of 1 mW. (d) Average temperature around the EO-Flex probe as a function of time for continuous (left, max 0.14 °C) and 20 Hz pulsed with 1 Hz on/off cycling (right, max 0.08 °C) of 470 nm light at a power of 208 μW.

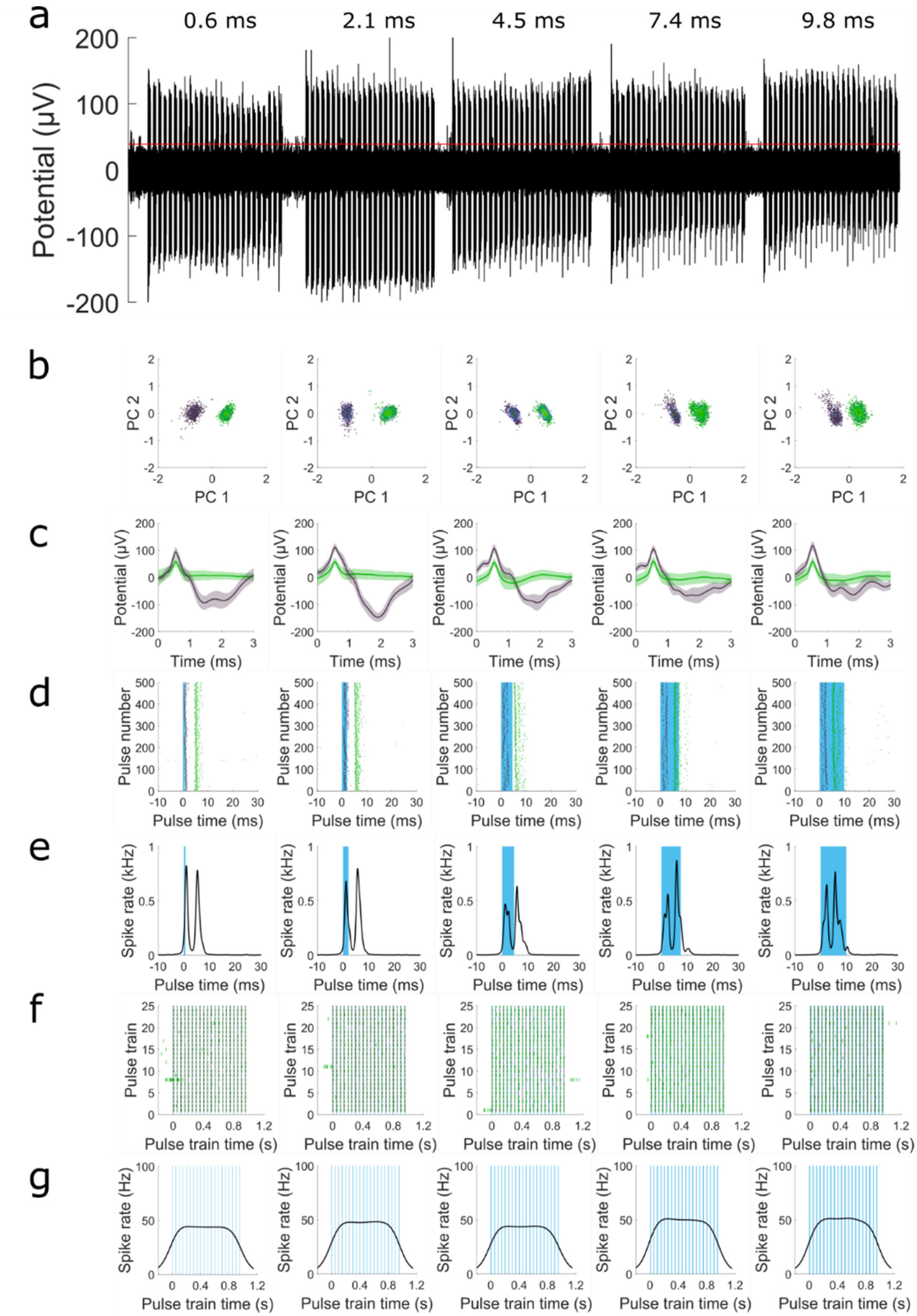
a
0.6 ms
2.1 ms
4.5 ms
7.4 ms
9.8 ms
Potential (μV)
b
PC 2
PC 1
c
Potential (μV)
Time (ms)
d
Pulse number
Pulse time (ms)
e
Spike rate (kHz)
Pulse time (ms)
f
Pulse train
Pulse train time (s)
g
Spike rate (Hz)
Pulse train time (s)

Figure S8. EO-Flex testing in layer 2/3 of a live Thy1-ChR2-YFP mouse as a function of the optical pulse width. All other stimulation parameters were held constant (optical stimulation power, 208 μW; stimulation frequency, 20 Hz; on/off cycling, 1 Hz). The recording depth was ~250 μm. (a) Optically evoked neural activity using pulse widths ranging from 0.6 ms to 9.8 ms. (b) The first two PCs of respective electrical recordings plotted with the Calinski-Harabasz metric for determining the number of clusters for mixed Gaussian fitting. (c) Average waveform (solid line) for each cluster from (b) with the shaded region representing one standard deviation. (d) Peri-stimulus plots for all optical pulses with spikes color-coordinated with the cluster from which they come. (e) Bayesian adaptive kernel smoother (BAKS) estimation of firing rate over the time window around the optical pulses. (f) Peri-stimulus plot for each optical pulse train as a function of the optical pulse width. (g) BAKS estimation for the firing rate over the pulse train window.

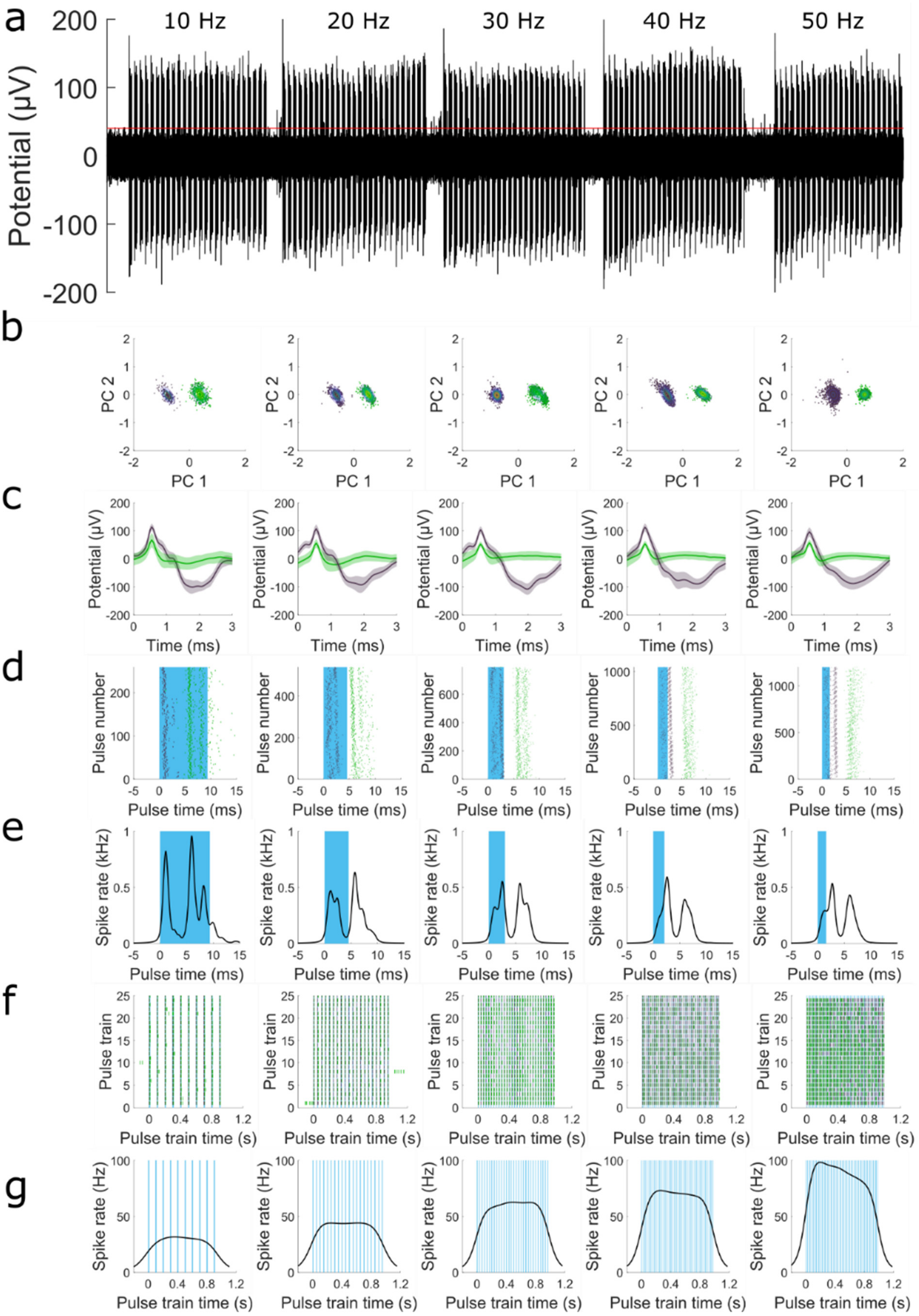
a
10 Hz
20 Hz
30 Hz
40 Hz
50 Hz
Potential (μV)
200
100
0
-100
-200
b
PC 2
PC 1
c
Potential (μV)
Time (ms)
d
Pulse number
Pulse time (ms)
e
Spike rate (kHz)
Pulse time (ms)
f
Pulse train
Pulse train time (s)
g
Spike rate (Hz)
Pulse train time (s)

Figure S9. EO-Flex testing in layer 2/3 of a live Thy1-ChR2-YFP mouse as a function of stimulation frequency. All other stimulation parameters were held constant (optical stimulation power, 208 μW; duty cycle, 10%; on/off cycling, 1 Hz). The recording depth was ~250 μm. (a) Optically evoked neural activity using stimulation frequencies ranging from 10 Hz to 50 Hz. (b) The first two PCs of respective electrical recordings plotted with the Calinski-Harabasz metric for determining the number of clusters for mixed Gaussian fitting. (c) Average waveform (solid line) for each cluster from (b) with the shaded region representing one standard deviation. (d) Peri-stimulus plots for all optical pulses with spikes color-coordinated with the cluster from which they come. (e) Bayesian adaptive kernel smoother (BAKS) estimation of the firing rate over the short time window around the optical pulses. (f) Peri-stimulus plot for each optical pulse as a function of the stimulation frequency. (g) BAKS estimation for the firing rate over the pulse train window.

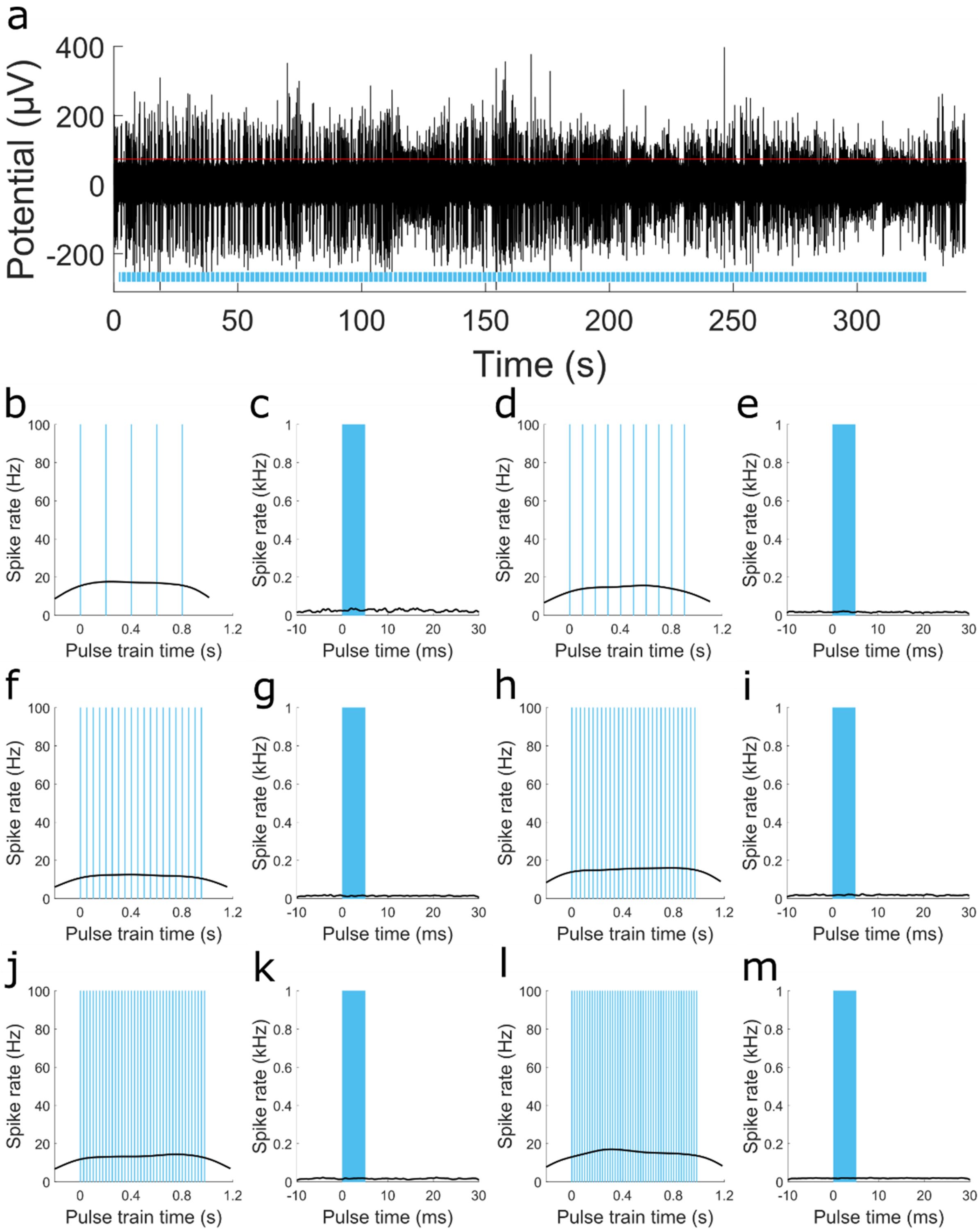

a
Potential (μV)
Time (s)
b
c
d
e
f
g
h
i
j
k
l
m
Spike rate (Hz)
Spike rate (kHz)
Pulse train time (s)
Pulse time (ms)

Figure S10. EO-Flex optical testing as a function of different optical stimulation frequencies (5, 10, 20, 30, 40, and 50 Hz) in an awake mouse without light-activated protein expression. All other stimulation parameters were held constant (i.e., optical stimulation power, 200 μW; pulse width, 4.5 ms; on/off cycling, 1 Hz). (a) Electrical recording (black) using a 50 Hz optical stimulus (blue). (b-m) Bayesian adaptive kernel smoother (BAKS) estimation for the firing rate over the pulse train window (left) and the BAKS firing rate for the individual pulses (right) for an optical stimulation frequency of 5 Hz (b-c), 10 Hz (d-e), 20 Hz (f-g), 30 Hz (h-i), 40 Hz (j-k), and 50 Hz (l-m).

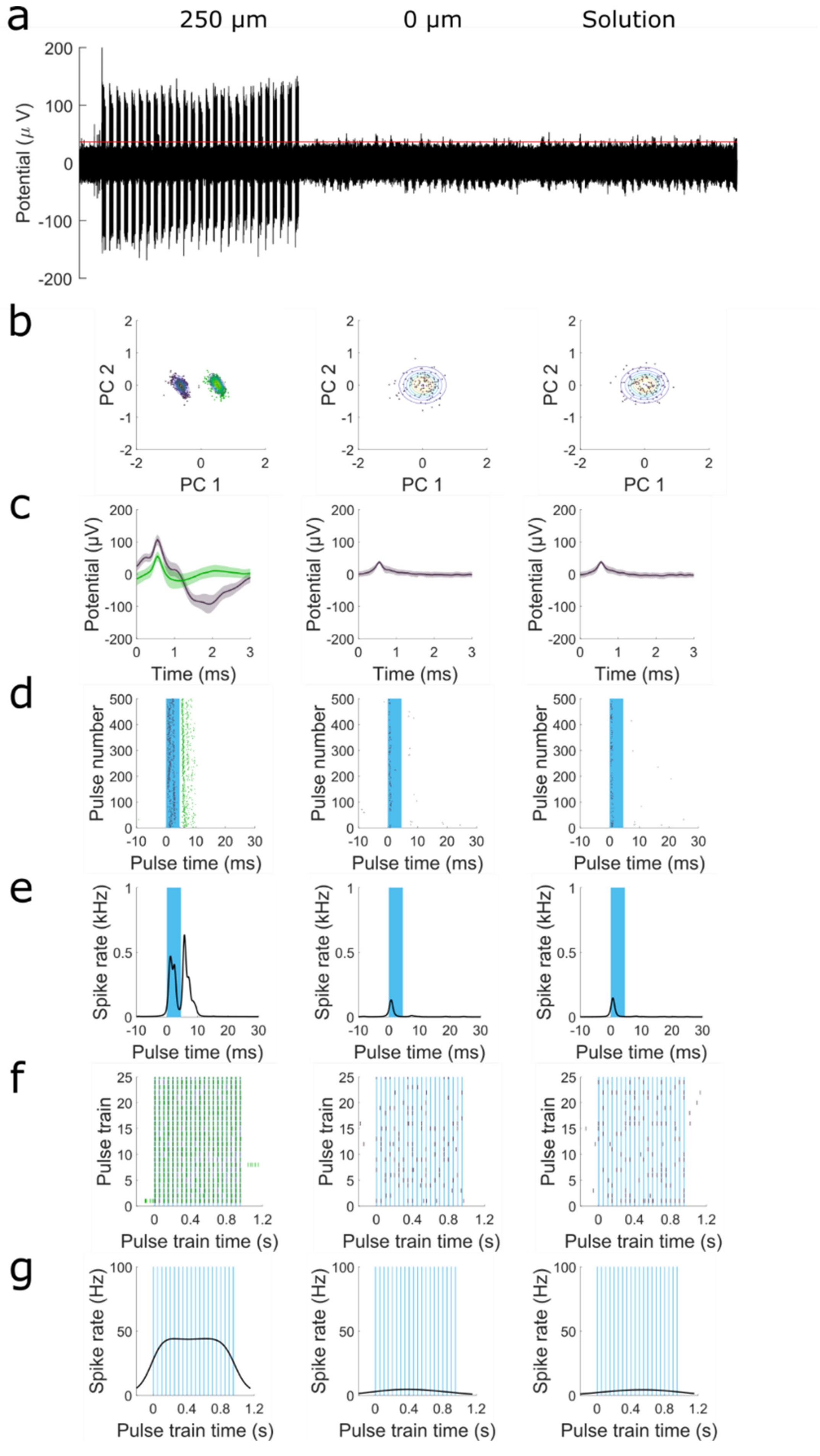
a
250 μm
0 μm
Solution
Potential (μ V)
b
PC 2
PC 1
c
Potential (μV)
Time (ms)
d
Pulse number
Pulse time (ms)
e
Spike rate (kHz)
Pulse time (ms)
f
Pulse train
Pulse train time (s)
g
Spike rate (Hz)
Pulse train time (s)

Figure S11. EO-Flex testing in a live Thy1-ChR2-YFP mouse as a function of recording depth. All other stimulation parameters were held constant (optical stimulation power, 208 μW; stimulation frequency, 20 Hz; pulse width, 4.5 ms; on/off cycling, 1 Hz). Recording depths were ~250 μm, 0 μm (i.e., at the agarose/brain interface), and in the saline solution above the craniotomy. (a) Optically evoked activity at different depths. (b) The first PCs of respective electrical recordings plotted with the Calinski-Harabasz metric for determining the number of clusters for mixed Gaussian fitting. (c) Average waveform (solid line) for each cluster from (b) with the shaded region representing one standard deviation. (d) Peri-stimulus plots for all optical pulses with spikes color-coordinated with the cluster from which they come. (e) Bayesian adaptive kernel smoother (BAKS) estimation of the firing rate over the short time window around the optical pulses. (f) Peri-stimulus plot for each optical pulse train for the different recording depths. (g) BAKS estimation for the firing rate over the pulse train window.

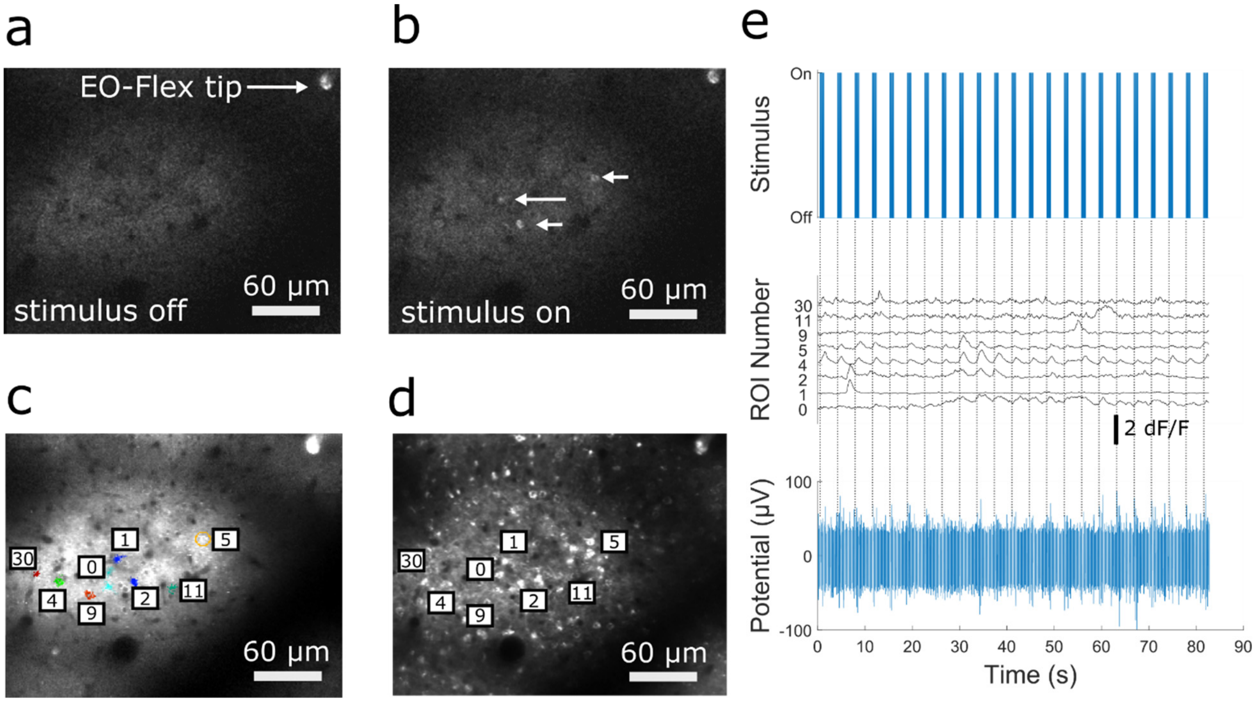

Figure S12. In vivo calcium imaging combined with simultaneous electrical recordings in the cortex of AAV2-CaMKII-C1V1-mCherry injected Vglut2-GCaMP6f mice confirms EO-Flex mediated optical excitation of neurons. (a-b) Fluorescence images from a time-lapse recording showing green fluorescent calcium indicator expressing neurons in layer 2/3 (depth, 270 μm) of a Vglut2-GCaMP6f transgenic mouse before (a) and immediately after (b) optical pulse train delivery to AAV2-CaMKII-C1V1-mCherry transduced cells. The probe tip is visible in the upper right corner of the field of view. The arrows in (b) indicate neurons that responded with fluorescence calcium transients to the optical pulses from the EO-Flex probe. (c) Average fluorescence image showing GCaMP6f expressing cells overlaid with regions of interest (ROIs) active during the 80 s stimulation period. Active ROIs were identified by automated analysis of cellular calcium signals using Suite2p (see Methods). (d) Average fluorescence image showing AAV2-CaMKII-C1V1-mCherry transduced neurons within the same field of view as in (c), confirming opsin expression in cells that show time-locked calcium responses to delivered optical pulses. (e) Successful optical excitation of neural activity was confirmed by simultaneous electrical recordings with the EO-Flex probe. (top) Delivered optical pulses (stimulation frequency, 8 Hz; pulse width, 12.2 ms; on/off cycling, 1 Hz) using 600 μW of power at the probe tip. (center) Calcium transients within the individual ROIs indicated in panel (c). (bottom) Recorded multi-unit activity after eliminating Becquerel effect mediated artifacts, caused by transient scanning of the imaging beam across the probe tip. Dashed lines are added for each pulse train to aid in the visual correlation between delivered optical pulses and measured calcium spiking and electrical activity. Simultaneous imaging, optical stimulation, and electrode recording experiments were performed with a single probe over multiple runs.

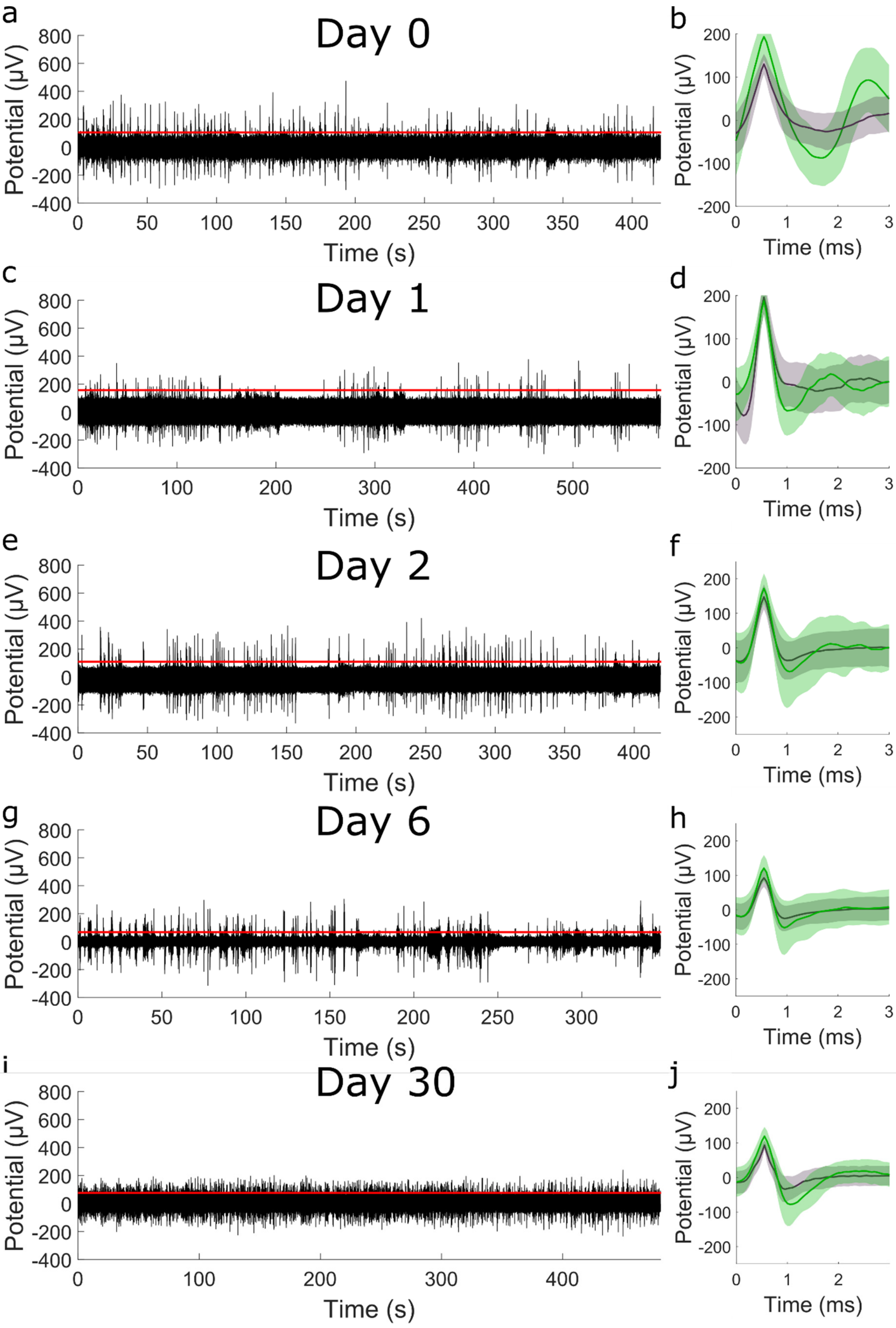
a
Day 0
b
c
Day 1
d
e
Day 2
f
g
Day 6
h
i
Day 30
j
Potential (μV)
Time (s)
Time (ms)

Figure S13. Chronic recordings with EO-Flex probes. (a-j) Spontaneous activity (left) and corresponding spike-sorted average waveforms with shaded one standard deviation (right) acquired with the same EO-Flex probe at different time points up to 1 month after implantation into the barrel cortex.

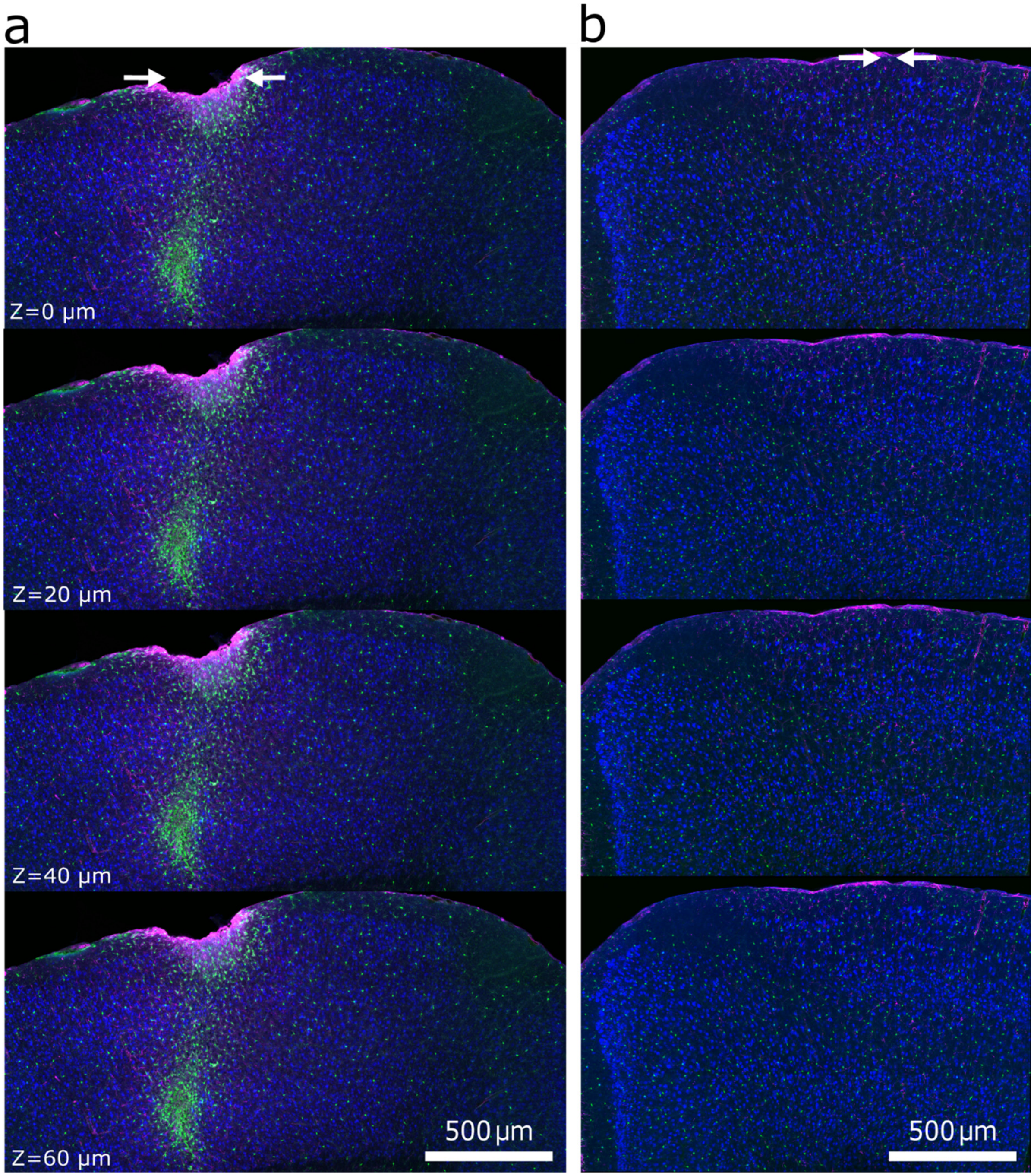

Figure S14. EO-Flex probes evoke minimal tissue inflammatory responses at 6 days post implant when compared to multimode fibers commonly used in optogenetic experiments. (a-b) Example images of 20 μm thick serial coronal brain sections around the multimode fiber (a) and EO-Flex probe (b) implantation sites (boundaries indicated by white arrows). Both the multimode fiber (diameter, 250 μm) and EO-Flex probe (diameter, 12 μm) were advanced to ~1 mm depth into the cortex. Images were taken one week after brain implantation in heterozygous Cx3cr1-GFP mice with labeled microglia (green). The sections were co-stained with anti-NeuN (blue) and anti-GFAP (magenta) antibodies to label neurons and astrocytes, respectively. z denotes slice spacing in microns. Results were reproduced across two different animals at 6 days post implant.

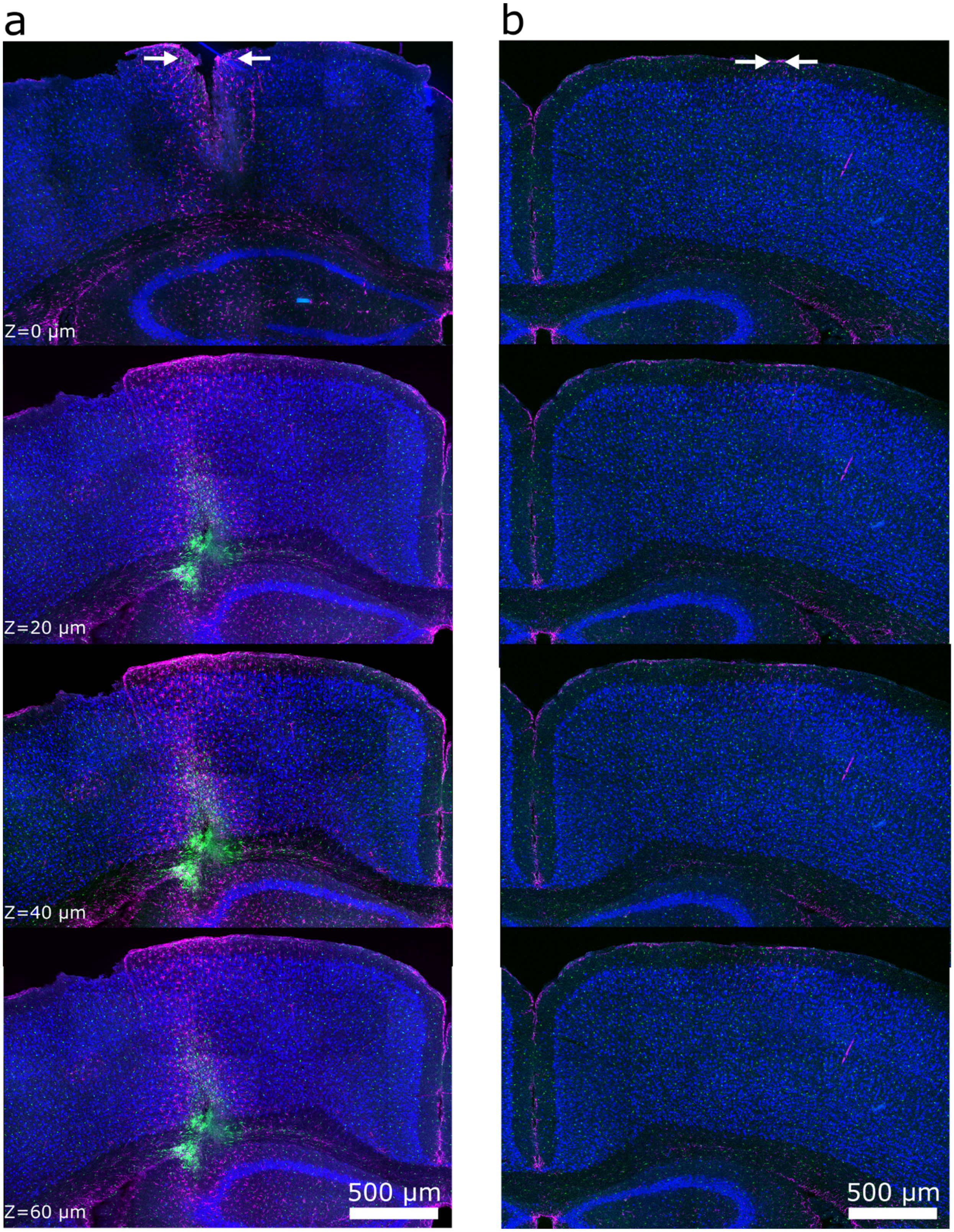
a
b
Z=0 µm
Z=20 µm
Z=40 µm
Z=60 µm
500 µm
500 µm

Figure S15. EO-Flex probes evoke minimal tissue inflammatory responses at 30 days post implant when compared to larger multimode fibers commonly used in optogenetic experiments. (a-b) Example images of 20 μm thick serial coronal brain sections around the multimode fiber (a) and EO-Flex probe (b) implantation sites (boundaries indicated by white arrows). Both the multimode fiber (diameter, 250 μm) and EO-Flex probe (diameter, 12 μm) were advanced to ~1 mm depth in the cortex. Images were taken one month after implantation in heterozygous Cx3cr1-GFP mice with labeled microglia (green). The sections were co-stained with anti-NeuN (blue) and anti-GFAP (magenta) antibodies to label neurons and astrocytes, respectively. z denotes slice spacing in microns. Results were reproduced across two different animals at 30 days post implant.

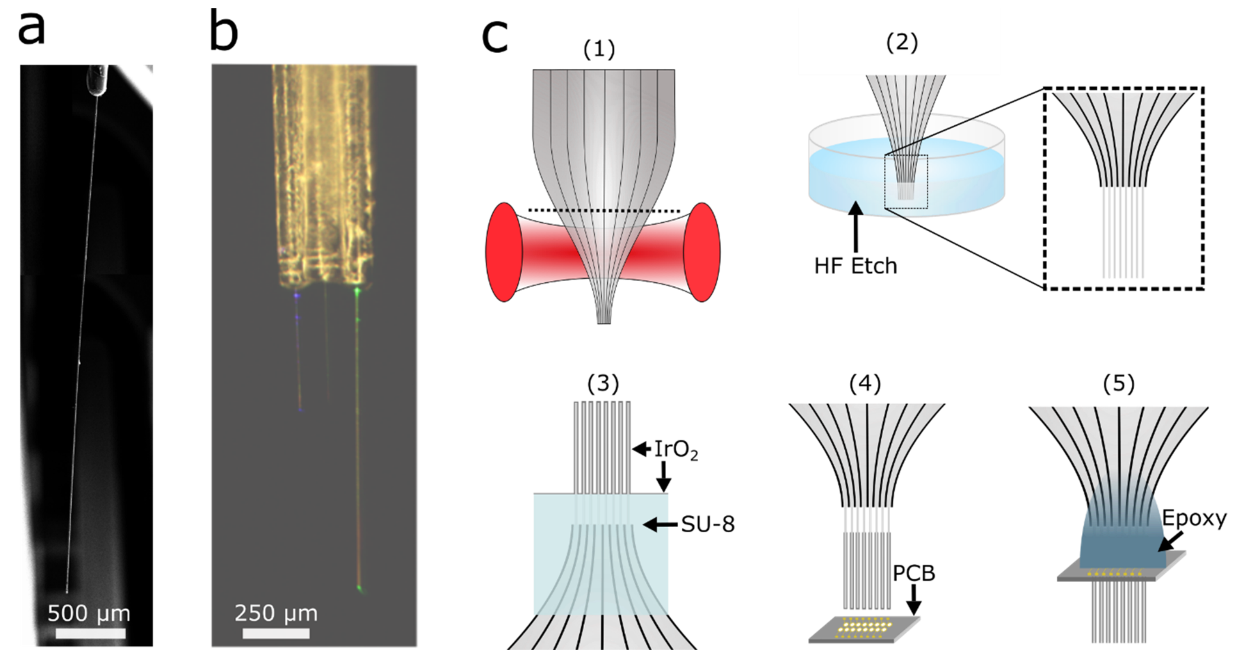

Figure S16. Scaling strategies of the EO-Flex probes for both probe length and arrays. (a) Two electron micrographs overlaid to show a 3.7 mm probe using a longer microfiber core. (b) Image showing a 3x1 EO-Flex array with individually addressable optical channels (442nm and 543nm output). (c) A proposed method for scaling probes into large two- or three-dimensional arrays (number of probes >100). (1) Utilize the same heat and pull strategy used to fabricate fiber bundles. Cut bundle at a desired backend diameter near the dashed line. (2) Use an acid bath to etch the surrounding cladding on the individual fiber cores with the length determined by the desired insertion depth. (3) Invert the etched structure and mask the bottom portion of the device to ensure electrical channels remain separate, then deposit the desired metal cladding layer(s). (4) Insert the array into a printed circuit board (PCB) with spacings tuned for the desired probe spacing/density that also allows tolerance for the insertion process (e.g., ~50 µm). (5) After inserting the array into the PCB, individual electrical connections are made using, for example, pin-in-paste reflow soldering techniques. The final assembly would use an adhesive to form a stable mechanical interface between the fiber bundle and PCB. The PEDOT:PSS and Parylene-C layers could then be deposited as previously described. Images of long and parallelized probes were taken once.

References

1. Dai, Z. R., Pan, Z. W. & Wang, Z. L. Novel nanostructures of functional oxides synthesized by thermal evaporation. *Adv. Funct. Mater.* **13**, 9–24 (2003).
2. Watakabe, A. Comparative molecular neuroanatomy of mammalian neocortex: what can gene expression tell us about areas and layers? *Dev. Growth & Differ.* **51**, 343–354 (2009).
3. Foutz, T. J., Arlow, R. L. & McIntyre, C. C. Theoretical principles underlying optical stimulation of a channelrhodopsin-2 positive pyramidal neuron. *J. Neurophysiol.* **107**, 3235–3245 (2012).
4. Stujenske, J. M., Spellman, T. & Gordon, J. A. Modeling the spatiotemporal dynamics of light and heat propagation for in vivo optogenetics. *Cell Rep.* **12**, 525–534 (2015).
