# Supplementary material for: Electro-optical mechanically flexible coaxial microprobes for minimally invasive interfacing with intrinsic neural circuits
Source: Nat Commun. 2022 Jun 7;13:3286. doi: 10.1038/s41467-022-30275-x (PMC9174211; doi:10.1038/s41467-022-30275-x)
Supplement: Supplementary file 4 — Reporting Summary [file 41467_2022_30275_MOESM4_ESM.pdf]

nature portfolio

Corresponding author(s): Donald Sirbuly, Axel Nimmerjahn

Last updated by author(s): Mar 21, 2022

Reporting Summary

Nature Portfolio wishes to improve the reproducibility of the work that we publish. This form provides structure for consistency and transparency in reporting. For further information on Nature Portfolio policies, see our Editorial Policies and the Editorial Policy Checklist.

Statistics

For all statistical analyses, confirm that the following items are present in the figure legend, table legend, main text, or Methods section.

n/a	Confirmed	
☐	☒	The exact sample size (*n*) for each experimental group/condition, given as a discrete number and unit of measurement
☐	☒	A statement on whether measurements were taken from distinct samples or whether the same sample was measured repeatedly
☐	☒	The statistical test(s) used AND whether they are one- or two-sided<br>*Only common tests should be described solely by name; describe more complex techniques in the Methods section.*
☒	☐	A description of all covariates tested
☐	☒	A description of any assumptions or corrections, such as tests of normality and adjustment for multiple comparisons
☐	☒	A full description of the statistical parameters including central tendency (e.g. means) or other basic estimates (e.g. regression coefficient) AND variation (e.g. standard deviation) or associated estimates of uncertainty (e.g. confidence intervals)
☐	☒	For null hypothesis testing, the test statistic (e.g. *F*, *t*, *r*) with confidence intervals, effect sizes, degrees of freedom and *P* value noted<br>*Give P values as exact values whenever suitable.*
☐	☒	For Bayesian analysis, information on the choice of priors and Markov chain Monte Carlo settings
☒	☐	For hierarchical and complex designs, identification of the appropriate level for tests and full reporting of outcomes
☒	☐	Estimates of effect sizes (e.g. Cohen's *d*, Pearson's *r*), indicating how they were calculated

Our web collection on statistics for biologists contains articles on many of the points above.

Software and code

Policy information about availability of computer code

Data collection	Electrical impedance spectroscopy data was recorded using VersaStudio (v.2.60.6) software.<br>Focused ion beam/scanning electron microscopy images were acquired with FEI Quanta FEG 250.<br>Optical micrographs taken during probe fabrication were recorded by Amscope (v3.7.9229.20170607).<br>Confocal image data were acquired using ZEN Black (Zeiss; v2011).<br>Two-photon imaging data were collected using MPScope software (Kleinfeld lab, UCSD).<br>Analog data were recorded using MPScope (Kleinfeld lab, UCSD) or DAQExpress 2.0 (National Instruments).<br>Video data were acquired using AVT SmartView 1.11 (Allied Vision Technologies).
Data analysis	Electrical and optical data were processed using custom MATLAB (v2019b) scripts (found here: https://github.com/Spencer-W/EO-Flex-Algorithms).<br><br>Bayesian Adaptive Kernel Smoother (BAKS 2017, v2a42835, https://github.com/nurahmadi/BAKS) was used for processing of spike rates in neural recording data.<br><br>Suite2p (found here: https://github.com/MouseLand/suite2p) was used for processing two-photon imaging data of neural activity, with fluorescence data being exported to .mat for use in a custom MATLAB (v2019b) script (found here: https://github.com/Spencer-W/EO-Flex-Algorithms).<br><br>Immune response images were processed, analyzed, and plotted using ImageJ (1.53f51), Imaris (Oxford Instruments, v 9.2), and Prism software (GraphPad Prism, v8.4.3).

For manuscripts utilizing custom algorithms or software that are central to the research but not yet described in published literature, software must be made available to editors and reviewers. We strongly encourage code deposition in a community repository (e.g. GitHub). See the Nature Portfolio guidelines for submitting code & software for further information.

Data

Policy information about availability of data

All manuscripts must include a data availability statement. This statement should provide the following information, where applicable:

- Accession codes, unique identifiers, or web links for publicly available datasets
- A description of any restrictions on data availability
- For clinical datasets or third party data, please ensure that the statement adheres to our policy

Source data are provided with this paper. Additional data that support the findings of this study are available from the corresponding authors upon reasonable request.

Field-specific reporting

Please select the one below that is the best fit for your research. If you are not sure, read the appropriate sections before making your selection.

☒ Life sciences ☐ Behavioural & social sciences ☐ Ecological, evolutionary & environmental sciences

For a reference copy of the document with all sections, see nature.com/documents/nr-reporting-summary-flat.pdf

Life sciences study design

All studies must disclose on these points even when the disclosure is negative.

Sample size	A sample size of n=4 probes was used for the in vivo evaluation of EO-Flex probe properties in this manuscript. Individual probes were utilized across up to n=2 different animal subjects for optical stimulation with electrical recording. Sample sizes for probes were based upon the availability of transgenic mice and fabricated probes. Additional probes (n=3) were used for long-term recordings and electrical stimulation. Immunostaining image analysis was based on n=8 brain sections per animal and two animals per time point (6 and 30 days). Sample size for brain sections were determined by the minimum slice thickness for handling purposes.
Data exclusions	Only data for which technical malfunction (e.g., optical shutter or tissue staining issues) occurred were excluded.
Replication	Individual probes were tested on up to two different animal subjects. The experiments yielded concomitant activity between optic stimulus and neural activity. Within each experiment, multiple runs (n>10) of successful optical stimulation and electrical recording were performed using the EO-Flex probes.
Randomization	Adult mice of both genders were randomly allocated to experimental groups.
Blinding	The investigators were not blinded to outcome assessment because they needed to record the identifier of the probe, sample, or animal.

Reporting for specific materials, systems and methods

We require information from authors about some types of materials, experimental systems and methods used in many studies. Here, indicate whether each material, system or method listed is relevant to your study. If you are not sure if a list item applies to your research, read the appropriate section before selecting a response.

Materials & experimental systems

n/a	Involved in the study	
☐	☒	Antibodies
☒	☐	Eukaryotic cell lines
☒	☐	Palaeontology and archaeology
☐	☒	Animals and other organisms
☒	☐	Human research participants
☒	☐	Clinical data
☒	☐	Dual use research of concern

Methods

n/a	Involved in the study	
☒	☐	ChIP-seq
☒	☐	Flow cytometry
☒	☐	MRI-based neuroimaging

Antibodies

Antibodies used	Primary antibodies included anti-GFAP (mouse monoclonal, clone GA5; EMD Millipore; cat. #MAB3402; RRID: AB_94844; 1:250 dilution) and anti-NeuN (rabbit polyclonal; EMD Millipore; cat. #ABN78; RRID: AB_10807945; 1:100 dilution). Secondary antibodies (1:100) included Alexa Fluor 405 goat anti-rabbit (Thermo Fisher Scientific; cat. #A-31556; RRID: AB_221605) and Alexa Fluor 633 goat anti-mouse (Thermo Fisher Scientific; cat. #A-21052; RRID: AB_2535719).
Validation	Validation according to manufacturers website for:

Validation	anti-GFAP (EMD Millipore; cat. #MAB3402); "Quality assurance is routinely evaluated by Western Blot on mouse brain lysate. Western Blot analysis of 1:1000 dilution of this lot detected Glial Fibrillary Acidic Protein on 10 µg of mouse brain lysate."<br><br>anti-Neun (EMD Millipore; cat. #ABN78); "Quality assurance is evaluated by Western Blotting in mouse E16 brain lysate. Western Blotting analysis: 0.5 µg/mL of this antibody detected NeuN in mouse E16 brain lysate."<br><br>AlexaFluor405 (Thermo; cat. #A-31556); "absorption 401nm; emission 420nm; microscopy (immunocytochemistry): Good nuclear staining, negligible background; purity(TLC): negligible or no free dye attached"<br><br>AlexaFluor633 (Thermo; cat. #A-31556); "absorption 630nm; emission 648nm; microscopy (immunocytochemistry): Good staining, negligible background; purity(TLC): negligible or no free dye attached"

Animals and other organisms

Policy information about studies involving animals; ARRIVE guidelines recommended for reporting animal research

Laboratory animals	For combined optogenetic and electrophysiological experiments, we used Thy1-ChR2-YFP male mice (stock #007612; Jackson Laboratories; age: 10 months); for combined calcium imaging, optogenetics, and electrophysiological experiments, we used AAV2-CaMKII-C1V1-mCherry-injected Vglut2-GCaMP6f male mice (a custom cross between Vglut2-Cre knock-in and Ai95D mice; stock #028863 and #024105, respectively; Jackson Laboratories; age: 3 months); for immune response and all other studies, we used heterozygous Cx3cr1-GFP male mice (stock #005582; Jackson Laboratories; age: 9 weeks).
Wild animals	The study did not involve wild animals.
Field-collected samples	The study did not involve animals collected from the field.
Ethics oversight	All live animal procedures were performed following the guidelines of the National Institutes of Health (NIH) and were approved by the Institutional Animal Care and Use Committee (IACUC) at the Salk Institute under protocol number 13-00022.

Note that full information on the approval of the study protocol must also be provided in the manuscript.
